# American trees shift their niches when invading Western Europe: evaluating invasion risks in a changing climate

**DOI:** 10.1002/ece3.2376

**Published:** 2016-09-22

**Authors:** Etienne Camenen, Annabel J. Porté, Marta Benito Garzón

**Affiliations:** ^1^ Biogeco INRA Université de Bordeaux 33615 Pessac France

**Keywords:** Climate change, niche equivalence, niche overlap, niche similarity, North American invasive trees

## Abstract

Four North American trees are becoming invasive species in Western Europe: *Acer negundo*,* Prunus serotina*,* Quercus rubra*, and *Robinia pseudoacacia*. However, their present and future potential risks of invasion have not been yet evaluated. Here, we assess niche shifts between the native and invasive ranges and the potential invasion risk of these four trees in Western Europe. We estimated niche conservatism in a multidimensional climate space using niche overlap Schoener's *D*, niche equivalence, and niche similarity tests. Niche unfilling and expansion were also estimated in analogous and nonanalogous climates. The capacity for predicting the opposite range between the native and invasive areas (transferability) was estimated by calibrating species distribution models (SDMs) on each range separately. Invasion risk was estimated using SDMs calibrated on both ranges and projected for 2050 climatic conditions. Our results showed that native and invasive niches were not equivalent with low niche overlap for all species. However, significant similarity was found between the invasive and native ranges of *Q. rubra* and *R. pseudoacacia*. Niche expansion was lower than 15% for all species, whereas unfilling ranged from 7 to 56% when it was measured using the entire climatic space and between 5 and 38% when it was measured using analogous climate only. Transferability was low for all species. SDMs calibrated over both ranges projected high habitat suitability in Western Europe under current and future climates. Thus, the North American and Western European ranges are not interchangeable irrespective of the studied species, suggesting that other environmental and/or biological characteristics are shaping their invasive niches. The current climatic risk of invasion is especially high for *R. pseudoacacia* and *A. negundo*. In the future, the highest risks of invasion for all species are located in Central and Northern Europe, whereas the risk is likely to decrease in the Mediterranean basin.

## Introduction

Exotic trees have been broadly planted for economic and ornamental purposes all over the world. However, in some cases, those trees became invasive, spreading quickly in their new niches. One specific case is that of four northeastern American tree species: red oak (*Quercus rubra* L.), black locust (*Robinia pseudoacacia* L.), black cherry (*Prunus serotina* Ehrh.), and boxelder maple (*Acer negundo* L.), that share most of their native ranges in North America and that are spreading quickly in Western Europe in different habitats. We would therefore hypothesize that those species would potentially share their climatic niche between the east coast of North America, where the core of these species distributions is located, and Western Europe, where they are actually spreading.

These trees were mostly introduced into Europe during the 17th and 18th centuries for ornamentation and forestry purposes (Lowe et al. 2000; Starfinger et al. 2003; Medrzycki 2007; Richardson and Rejmánek 2011; Cierjacks et al. 2013; Woziwoda et al. 2014), and some of them still have an economic importance. For example, since the beginning of the 20th century, *Q. rubra* and *R. pseudoacacia* have benefited from tree breeding programs for improving their growth and fruit production, mainly in Central Europe (Keresztesi 1983; Kremer 1986, 1994; Rédei et al. 2008). Until the 1950s, *P. serotina* was also used in forest plantations in Northern Europe, while *Q. rubra* and *R. pseudoacacia* are still largely used for forestry purposes or erosion control. *Acer negundo* is still largely planted in urban areas for ornamentation purposes. Their impacts have not yet been fully evaluated, but loss of local biodiversity, forest regeneration difficulties, river bank collapse, and changes in soil properties have already been identified in several countries. Some of these species as *R. pseudoacacia* and *P. serotina* are included in the 100 worst invasive species (Hulme 2009). Their impacts have not yet been fully evaluated, but loss of local biodiversity, forest regeneration difficulties, river bank collapse, and changes in soil properties have already been identified in several countries, which highlights the current conflict between foresters and managers of natural areas (Dickie et al. 2014).

Invasive species and climate change are considered to be equally important major causes of biodiversity loss after the destruction of direct natural habitat (Corvalan et al. 2005). Although new climates should impact biological invasions in the near future (e.g., Hellmann et al. 2008; Walther et al. 2009), there is little empirical evidence on the direction and intensity of this impact. For instance, the flowering phenology of invasive plants was demonstrated to track the temperature increase from 1900 to 2006 better than that of non‐native and native plants of a North American forest (Willis et al. 2010), which could favor their development and reproduction. The increase in extreme climatic events such as heat waves or storms could favor exotic species by facilitating propagule dispersal, creating empty niches, and releasing native species competition (Diez et al. 2012). On the other hand, suitable habitats for invasive species are expected to decrease under future climate change, but models show very high interspecific variability (e.g.*,* Peterson et al. 2008; Bellard et al. 2012). Therefore, individual case studies are required to give a clearer picture of the suitable habitats and risk of invasion for many invasive species.

Species distribution models (SDMs) are used to assess the suitable habitat for a given species, which in the case of invasive ranges relates to the invasibility of the species. To evaluate the differences in native and invasive niches, several tests have been developed (Broennimann et al. 2007, 2012; Warren et al. 2008; Petitpierre et al. 2012; Guisan et al. 2014; Aguirre‐Gutiérrez et al. 2015). Most of these are calculated from a multivariate climatic space that applies kernel smoothers to the species occurrences, allowing to check for niche conservatism (Warren et al. 2008): The niche *overlap test* measures the degree of overlap between two niches (Schoener 1968; Warren et al. 2008), the *equivalence test* measures whether one species distribution is identical to another, and the *niche similarity* is an estimate of the degree of niche overlap between two niches against the null distribution of *n* data randomly sampled in the climatic space of one niche (Warren et al. 2008; Aguirre‐Gutiérrez et al. 2015). Other tests have been specifically developed for the analysis of invasibility, attempting to estimate the degree of *niche expansion* (i.e., when the exotic niche is not overlapping with the native one) and *unfilling* (i.e., when native niche is not overlapping with the exotic one) in the invasive range of the species (Petitpierre et al. 2012; Guisan et al. 2014). Finally, different calibrations of SDMs using native, invasive, and both ranges together check for niche *transferability*, defined as the capacity of a model calibrated on one range to predict the climatic niche of the species in another (Fernández and Hamilton 2015).

We assessed potential shifts in the suitable habitats of four invasive trees in Western Europe. We first estimated the degree of niche overlap between North American and Western European ranges of the species using *niche overlap*,* niche equivalence*, and *niche similarity tests* based on a multiclimatic space defined for the native and invasive ranges of the species, and secondly using a *transferability range analysis* based on a combination of multiple calibrations of SDMs. Finally, we evaluated the climatic invasion risks in Western Europe under the present climate and that predicted for 2050 (Representative Concentration Pathway 4.5), running SDMs calibrated over both ranges of the species.

## Methods

### Data collection

#### Native and European invasive species ranges

The native ranges of the four species of interest correspond to those established by Little (1971), extracted from the USGS database ( http://esp.cr.usgs.gov/data/little/, accessed in February 2015). In Western Europe, their occurrence was established by compiling several data sources (see Table S1); data were collected with a resolution of less than or equal to 5′, and any data from earlier than 1950 (the beginning of the period with continuous climate data) were removed. To overcome the sampling heterogeneity of the data sources, a single occurrence point was extracted for each 10 × 10 km grid of a WGS projection when it contained more than one occurrence. In regions with well‐established spatial coverage (France, Spain, the United Kingdom, Flanders, the Netherlands, Germany, and Switzerland), “pseudo”‐absence data were obtained by applying a 30‐km buffer zone around each occurrence point. All grid points within the buffer area were considered as absences. “True” absences in Italian regions were also extracted from the database *Acta plantarum* ( www.actaplantarum.org, accessed in March 2015). All vector maps were rasterized in the GRASS GIS open source software (v.7.0.0; GRASS Development Team, 2015) and interpolated to 5 arc‐minutes resolution (≈10 × 10 km).

#### Climate data

Climate data were extracted from the WorldClim database ( www.worldclim.org), for the current climate (1950–2000) and for that of 2050 according to the intermediate RCP 4.5 scenario (IPCC, 2014), which supposes the stabilization of radiative forcing in 2100, resulting in moderate increases in CO_2_ (650 ppm; Moss et al. 2010) and temperature (1.1–2.6°C on average over the 21st century; Collins et al. 2013). The climate dataset used for this study was estimated as the average from 11 global circulation models (BCC‐CSM1‐1, CCSM4, GISS‐E2‐R, HadGEM2‐AO, HadGEM2‐ES, IPSL‐CM5A‐LR, MIROC5, MRI‐CGCM3, MIROC‐ESM‐CHEM, MIROC‐ESM, and NorESM1‐M) to reduce the uncertainty due to models over the period 2041–2060 (referred as “2050” hereafter). The coefficient of variation between models was low for all the variables used (Fig. S1).

Seven climatic variables were selected to build the SDMs (correlation between variables are shown in Table S1): temperature seasonality (TS), corresponding to the monthly standard deviation from the mean annual temperature; mean temperature of the warmest month (MTWM, °C); mean temperature of the coldest month (MTCM, °C); precipitation seasonality (PS), corresponding to the coefficient of variation of the mean annual precipitation; mean precipitation of the driest quarter (PDQ, mm), a quarter corresponding to three successive months; mean precipitation of the warmest quarter (PWQ, mm); and mean precipitation of the coldest quarter (PCQ, mm).

### Model calibrations

Species distribution models for all four species for North America and Western Europe were calibrated and projected under current and future climatic conditions. We use the term suitability index (SI) to refer to the probability of one species to inhabit a given location as predicted by the model. SI ranges from 0 (not suitable) to 1 (most suitable). We used the Random Forest algorithm (Breiman 2001; randomForest library R), a machine learning method that implements bootstrap aggregation of trees (bagging) for producing the best regressions for each model.

Three types of calibration were carried out to evaluate the transferability of the invasive and native ranges either using presence/absence data (*P*/*A*) from the native range of the species (SDMs‐NA) or *P*/*A* from Western Europe (SDMs‐EU). Calibration performed using data from the two areas (native and introduced ranges; SDMs‐NAEU) allowed evaluating climatically suitable areas in the invasive range. A similar proportion of *P*/*A* data was retained in each dataset to ensure a similar ratio of North American and European occurrences for the SDMs‐NAEU calibration. Due to datasets, true absences were used for calibrations in North America, while pseudo‐absences were used for calibrations in Europe. Each calibration was run 10 times to compensate for the variance effect on considering a subset of *P*/*A* sampling. The importance of each driver in the models was evaluated by the increase in the mean square error of the variance when the variable was omitted. The generalization power of the SDMs was estimated using Pearson's correlations between the predicted response of the test set and the observed data. Two‐thirds of the total dataset was used for calibration, one‐third (test set) to evaluate the goodness of fit of the models, independently. Goodness of fit was assessed using the percentage of the variance explained by the model and by comparison of the prediction maps with the observed ones using true skill statistics (TSS; Allouche et al. 2006) calculated as follows:

(1) $$\text{TSS}=\text{Se}+\text{Sp}-1;\;\text{where}\;\left\{\begin{array}{c} \text{Se}=\text{TP}/P\\ \text{Sp}=\text{TA}/A \end{array}.\right.$$

Here, Se (sensitivity) corresponds to the proportion of the observed presences predicted correctly (ratio of TP, number of true positives, to *P*, total number of presences) and Sp (specificity) corresponds to the proportion of observed absences predicted correctly (ratio of TA, number of true absences, to *A*, total number of absences). TSS varies between −1 and 1, from the worst to the best predictions. Because invasive species may not yet be fully settled in their invaded range, goodness of fit for Europe was evaluated using Se alone (varying from 0 to 1, with 1 indicating a perfect prediction; Guisan and Thuiller 2005).

### Testing niche overlap, niche equivalence, niche similarity, degree and direction of niche shift, and niche transferability

Two approaches were used to test niche shift between the native and invasive ranges. The first is based on a bidimensional climate space that applies kernel smoothers to the species occurrences (Broennimann et al. 2007, 2012; Warren et al. 2008; Guisan et al. 2014), and the second is based on multiple calibrations of SDMs (Fernández and Hamilton 2015). For the first approach, the climatic space for North America and Western Europe was defined following the terrestrial ecoregions of the world (Olson et al. 2001), including all North America and Western Europe biomes except Tundra. In each range, the climatic space was summarized using the first two axes of a principal component analysis (PCA) including seven climatic variables (TS, MTWM, MTCM, PS, PDQ, PWQ, and PCQ) gridded to a resolution of 100 × 100 climatic units as defined by the PCA axis. Occurrence data from the native and invasive ranges of the species were projected onto the climatic space of the PCA after converting it into a probability of occurrence (Guisan et al. 2014). Use of this climatic space facilitates the estimation of several niche conservatism indexes and tests, as described below.

The *niche overlap D* between both niches was calculated according to Schoener (1968):

(2) $$D=1-\frac{1}{2}\left(\sum_{ij}\left|z_{1,ij}-z_{2,ij}\right|\right)$$

where *z*_1,*ij*_ and z_2,*ij*_ correspond for each cell_*ij*_ to the probability of occurrence of the species corrected using the climatic availability in the background climatic space as used in the PCA in the native (1) and invasive (2) ranges, respectively (Warren et al. 2008; Broennimann et al. 2012). The value of *D* ranges from 0 to 1 from null to total niche overlap.

The *equivalence test* was used to assess whether the native and invasive ranges of the species were interchangeable (Warren et al. 2008; Aguirre‐Gutiérrez et al. 2015). Niche equivalence tests were performed by comparing the niche overlap statistics *D* estimated using the occurrences of the native (*S*_N_) and invasive (*S*_I_) species to the null distribution of 100 random replicates taken from the entire distribution (*S*_N_ + *S*_I_).

The *similarity test* was used to estimate the similarity of the climatic niche in the invasive range to that in the native one. Similarity test was performed by comparing the niche overlap *D* calculated between the native and invasive ranges against the null distribution of 100 datasets randomly sampled in the climatic space of the invasive range. A significant test result indicates that the observed overlap was significantly different from a random overlap and therefore that the invasive niche is not similar to the native one.

The degree and direction of niche shift were estimated by calculating the fraction of the climatic space occupied by the species only in its native range (*unfilling index*,* U*) and the fraction of the climatic space occupied by the species only in the invasive range (*extension index*,* E*; Petitpierre et al. 2012). This analysis was performed considering the whole climatic space of the species in both ranges and considering only the analogous climatic space between the two ranges. Low values of *U* and *E* indexes are interpreted as niche overlap; a high *E* index value is interpreted as a niche shift and a high *U* index value can also correspond to a shift, but can equally indicate an early invasion stage because the invasive species has not yet colonized its new territory.

Niche *transferability* is an estimate of the capacity of SDMs calibrated using the occurrence of the species in its native range (SDMs‐NA) to predict correctly the habitat suitability in the invasive range (SDMs‐EU) and vice versa (Fernández and Hamilton 2015). Transferability was evaluated using the TSS when testing transferability to the native range and using the sensitivity Se (equation (1)) when testing transferability to the invasive range. In addition, the SDMs‐NAEU transferability was similarly evaluated using a calibration performed over the two ranges and using predictions evaluated over each range separately.

**Table 1:** Main characteristics of the principal component analysis calibrated on the climatic space of the North American and Western European backgrounds

Total inertia (%)	Axis 1 42.3		Axis 2 71.7	
	Eigenvalues (%)	Correlation	Eigenvalues (%)	Correlation
PDQ	**25.4**	0.87	8.6	−0.42
PCQ	**21.6**	0.80	0.3	−0.07
TS	18.5	−0.74	16.4	−0.58
MTCM	14.9	0.66	**25.7**	0.73
PS	12.7	−0.61	7.6	0.40
PWQ	6.7	0.44	8.3	−0.41
MTWM	0.2	−0.07	**33.1**	0.82

Total cumulated inertia (%) of the two first axes is presented, and then eigenvalues of the climatic variables are indicated per axis. PDQ, precipitation of the driest quarter; PCQ, precipitation of the coldest quarter; TS, temperature seasonality; MTCM, mean temperature of the coldest month; PS, precipitation seasonality; PWQ, precipitation of the warmest quarter; MTWM, mean temperature of the warmest month. Bold characters indicate the main contributors to each axis component. All correlations were highly significant (*P* < 10^−5^).

## Results

### Characteristics of the climatic niches in the native and introduced ranges

**Figure 1:**
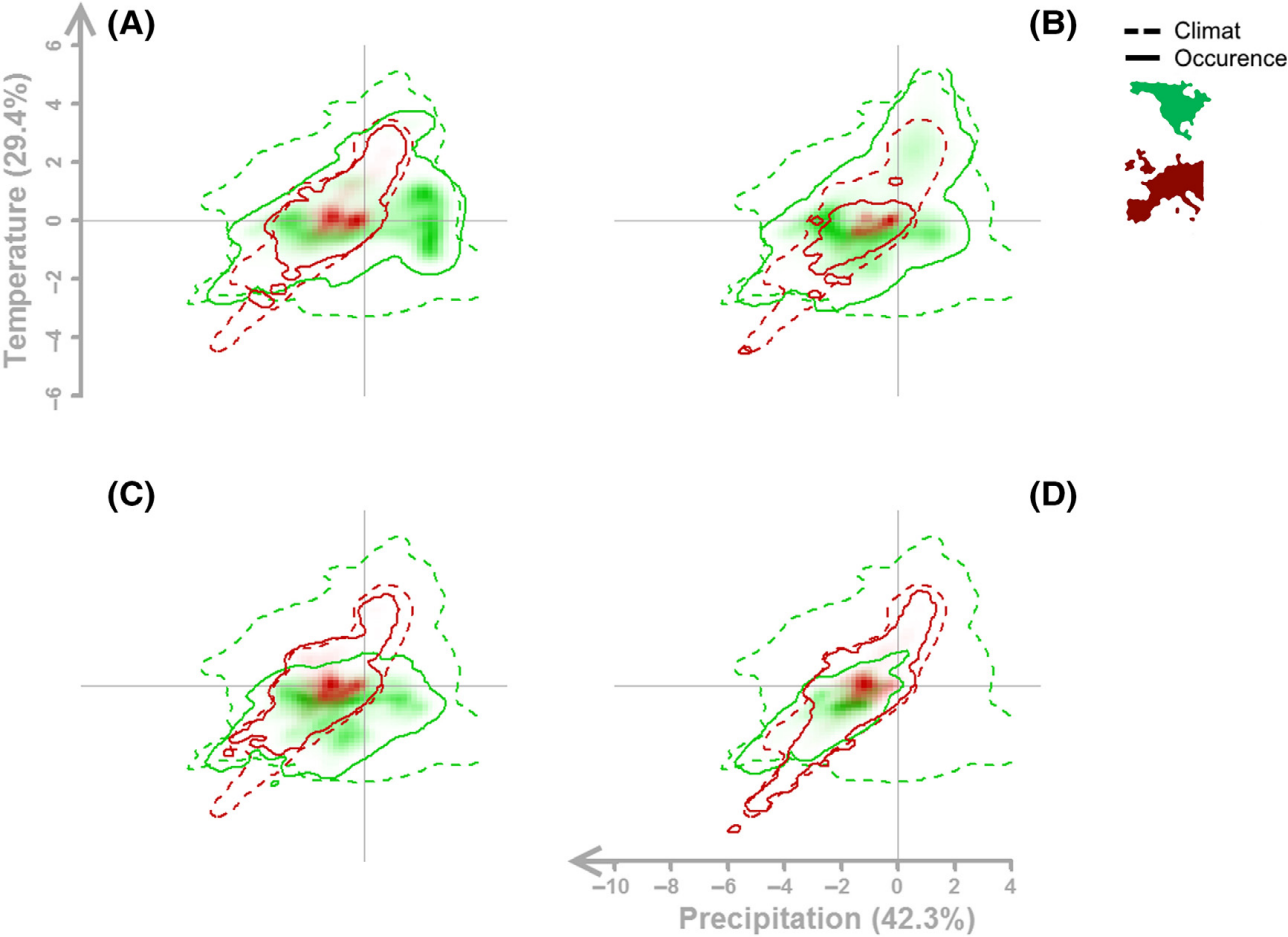
Climatic niches (full line) of the four species based on the 75% quantile of probability of occurrence as projected in the climatic space background (dotted line) of the native (green line) and introduced (red line) ranges. The climatic space is defined by the first two axes of the principal component analysis (PCA) presented in Table 1. Green and red shadings represent the centroid of the climatic niche based on occurrence data in the native and invasive ranges, respectively. (A) *Acer negundo*, (B) *Prunus serotina*, (C) *Quercus rubra*, (D) *Robinia pseudoacacia*. Climatic variables used in the PCA are MTCM, mean temperature of the coldest month; MTWM, mean temperature of the warmest month; PCQ, precipitation of the coldest quarter; PDQ, precipitation of the driest quarter; PS, precipitation seasonality; PWQ, precipitation of the warmest quarter; TS, temperature seasonality.

The climatic space of the North American and Western European backgrounds is represented in a PCA analysis (Fig. 1): The two first axes explain 71.7% of the total variance Table 1. The first axis was globally characterized by a negative precipitation gradient, and the second axis was characterized by a positive thermic gradient. The projections of the species niches in the native and invasive ranges (Fig. 1) were analyzed using the tests shown in Table 2. Schoener's *D* varied between 0.29 and 0.45, corresponding to weakly overlapping climatic niches between the invasive and native ranges. This was confirmed by the equivalence test results, which were significant irrespective of the species tested. Thus, the null hypothesis of equivalence was rejected in all cases. The *similarity test* was only significant for *Q. rubra* and *R. pseudoacacia*, indicating that the differences in niche between the invasive and native ranges were greater than those that might be expected by chance. Therefore, the differences in niche were not only related to the availability of the climatic conditions in the native range. Overall these tests showed that, although the niches were similar for *Q. rubra and R. pseudoacacia*, none of the species had an identical niche in their invasive and native ranges.

**Table 2:** Tests and indexes used for niche change analysis between the native and invasive ranges

Species	*D*	Eq. test	Sim. test	*E*_a_ (%)	*E*_na_ (%)	*U*_a_ (%)	*U*_na_ (%)
*Acer negundo*	0.43	**0.02**	0.01	0	0	5	56
*Prunus serotina*	0.29	**0.02**	0.05	0	0	38	54
*Quercus rubra*	0.45	**0.01**	**0.11**	6	6	8	41
*Robinia pseudoacacia*	0.39	**0.04**	**0.11**	7	12	6	7

Value of Schoener's *D* between both niches (varying from 0 for no overlap to 1 for a perfect niche overlap), equivalency (Eq. test; test's *P*‐value, with bold text indicating significance at *α* = 0.05) and similarity (Sim. test; test's *P*‐value, with bold text indicating significance at *α* = 0.05). Indexes of niche expansion (*E*, %) and niche unfilling (*U*, %) calculated considering the whole background climatic space (na) of the species from both ranges, or considering only the analogous climatic space (a) shared between both ranges.

Niche differences were mostly related to unfilling for *A. negundo*,* Q. rubra,* and *P. serotina*, with large unfilling for *P. serotina* in the analogous climate (*U*_a_ = 0.38). Niche expansion remained null for *A. negundo* and *P. serotina* and ranged from 6 and 12% for *Q. rubra* and *R. pseudoacacia* (Table 2).

Transferability, or the capacity of the SDMs to predict the presence of the species in another range, was poor when the calibration was performed on one range and tested against the other: Se varied from 0 to 0.07 for SDMs‐NA transferred to the invasive range and TSS for SDMs‐EU varied from −0.16 to 0.26 the other way round. The transferability maps clearly show that models calibrated on one range do not accurately predict the niche in the other (Figs. S1, S2). However, when the SDMs were calibrated over both ranges (SDMs‐NAEU), all indicators of goodness of fit were high (variance explained > 90%, Pearson's *r* > 0.96, TSS goodness of fit > 0.94; Table 3). Interestingly, the transferability of SDMs‐NAEU over either the native or the invasive range separately was high: TSS varied from 0.94 to 0.98 in North America, and Se was equal to 1.00 irrespective of species in Western Europe (Table 3).

**Table 3:** Evaluation of the performance of the SDMs: goodness of fit was evaluated as the percentage of variance explained by the models (PVE)

Species	PVE	*r*	Goodness of fit		NA transferability		EU transferability		Number of cases	
			TSS	SI	TSS	SI	Se	SI	Pres. (%)	Tot.
SDM‐NAEU										
*Acer negundo*	90.95	0.97*	0.94	0.83	0.94	0.79	1.00	0.52	22.6	30,1551
*Prunus serotina*	92.19	0.96*	0.94	0.84	0.94	0.86	1.00	0.56	18.8	30,2082
*Quercus rubra*	96.42	0.98*	0.96	0.89	0.97	0.88	1.00	0.51	16.5	30,4365
*Robinia pseudoacacia*	97.00	0.99*	0.97	0.99	0.98	0.99	1.00	0.51	4.9	30,8405
SDM‐NA										
*Acer negundo*	94.72	0.98*	0.98	0.88	–	–	0.07	0.5	22	29,9138
*Prunus serotina*	95.21	0.98*	0.98	0.91	–	–	0.02	0.5	18	29,9138
*Quercus rubra*	97.97	0.99*	0.99	0.94	–	–	0.01	0.5	15	29,9138
*Robinia pseudoacacia*	96.60	0.98*	0.96	1.00	–	–	0.00	0.51	2	29,9138
SDM‐EU										
*Acer negundo*	80.83	0.95*	0.97	0.5	0.26	0.67	–	–	41	5958
*Prunus serotina*	92.32	0.97*	0.99	0.5	−0.16	0.8	–	–	24	12,369
*Quercus rubra*	89.60	0.98*	0.99	0.5	0.10	0.5	–	–	53	9881
*Robinia pseudoacacia*	92.69	0.96*	0.97	0.5	0.09	0.93	–	–	90	10,285

**Figure 2:**
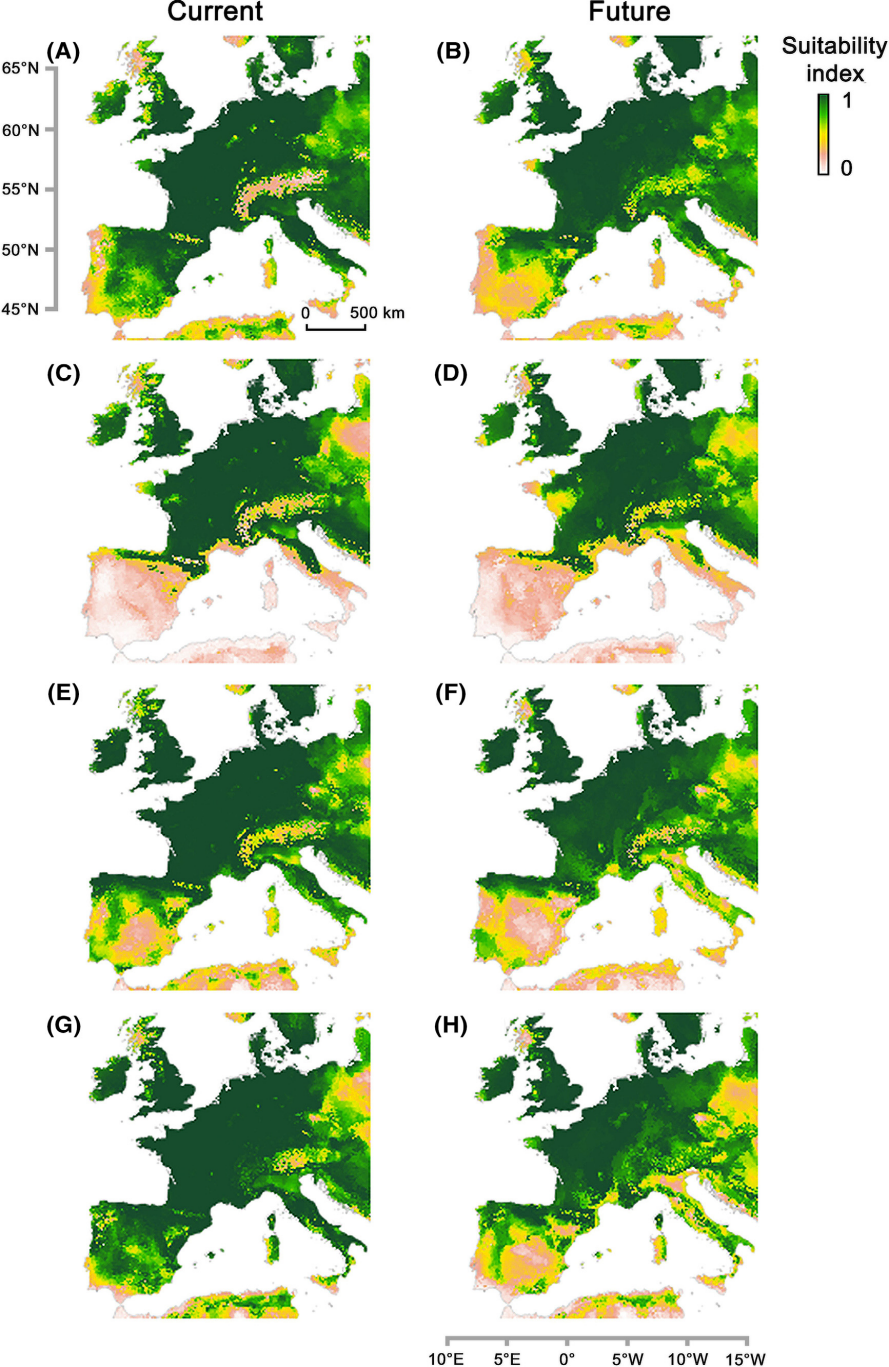
Habitat suitability in current and future climate conditions for the studied species calibrated on presence data in Western Europe (mainly France, Spain, the United Kingdom, Flanders, Switzerland, and Germany) and on *P*/*A* in the native range in North America: *Acer negundo* for current (A) and future periods (B), *Prunus serotina* for current (C) and future periods (D), *Quercus rubra* for current (E) and future periods (F), and *Robinia pseudoacacia* for current (G) and future periods (H). Dark green indicates high suitability, yellow indicates moderate suitability, and light red indicates low suitability.

Generalization power was assessed by Pearson's correlation r between observed *P*/*A* values and predicted values using an independent dataset (*, *P* < 0.05). The capacity of the SDM to predict the presence of the species was assessed using true skill statistics (“TSS”), ranging from −1 to 1 (from the worst possible to a perfect match between the habitat suitability prediction and the occurrence of the species). The suitability index threshold allows the conversion from SI continuous maps into binary maps. Maps are presented in Figure 2 and Figures S2 and S3. The values shown correspond to the SI threshold chosen to optimize TSS values. Values are averaged on the results of 10 SDM runs. SDMs‐NAEU were calibrated on presence data from Western Europe (mainly France, Spain, the United Kingdom, Flanders, Switzerland, and Germany) and presence/absence data from North America; SDMs‐NA were calibrated on *P*/*A* data from North America, and SDMs‐EU were calibrated on *P*/*A* from Western Europe. Total number of points used and percentage of plots where the species is present are indicated. PVE is the percentage of the variance explained by the models; *r* is the Pearson coefficient. TSS is the true skill statistics and SI the suitability index coming for the predictions. Se is the sensitivity. Pres., presences; Tot., total of presences + absences.

### Predicting species niches in Western Europe

#### Performance of the species distribution models

Overall, all SDMs calibrated using North American native distributions of the species (SDMs‐NA) and SDMs calibrated with Western European invasive ranges of the species (SDMs‐EU) presented a high goodness of fit: They explained from 95 to 97% and from 80 to 93% of the variance according to the species for SDMs‐NA and for SDMs‐EU, respectively (Table 3). Several algorithms were tested for these models (Table S2), but only SDMs obtained using the RF algorithm are presented here because this showed the best performance of all of the models (Table S3). The most important climatic predictors were similar for all the species (SDMs‐NAEU). In particular, the most important variables explaining the variance of the models were MTCM and MTWM, followed by PS and PWQ. TS, PDQ, and PCQ were, on average, less important variables in the model and showed more variability between species (Table S4).

#### Current and future suitable habitats in Western Europe for invasive North American trees

SDMs‐NAEU were used to characterize the suitable habitats in Western Europe under current and future climatic conditions (Fig. 2). Overall, Western Europe presented a high suitability for the four species in northern and temperate regions, whereas the SI was much lower in the Mediterranean region (Fig. 2, Table 4). The projections for 2050 showed a general northward shift of the suitable conditions for all species.

**Table 4:** Average suitability index (SI) per country or geographical region for current predictions (1950–2000) and for 2050 predictions (2040–2060) for each species estimated using the SDMs‐NAEU for both native and invasive range presence/absence data

Species	Period	BE	DE	FR	GE	IR	IT	NE	NO*	PO	SP	SWE*	SWI	UK	EU
*Acer negundo*	Current	0.98	0.90	0.87	0.99	0.81	0.79	0.99	**0.17**	**0.44**	**0.80**	**0.36**	**0.63**	**0.81**	0.85
	2050	0.99	0.96	0.87	0.98	0.89	0.73	0.99	**0.36**	**0.34**	**0.63**	**0.59**	**0.80**	**0.91**	0.83
*Prunus serotina*	Current	0.99	0.97	**0.83**	0.99	0.86	0.53	0.99	**0.14**	0.09	0.27	**0.32**	0.72	0.84	0.72
	2050	0.99	0.90	**0.76**	0.99	0.84	0.46	0.99	**0.30**	0.07	0.26	**0.51**	0.80	0.88	0.7
*Quercus rubra*	Current	1.00	0.96	0.89	0.99	0.97	**0.75**	0.99	**0.19**	0.56	**0.65**	**0.33**	0.73	0.93	0.84
	2050	0.99	0.93	0.84	0.96	0.96	**0.60**	0.99	**0.32**	0.50	**0.49**	**0.56**	0.72	0.92	0.78
*Robinia pseudoacacia*	Current	1.00	0.96	0.90	0.99	0.93	**0.84**	0.99	**0.18**	**0.71**	**0.83**	**0.27**	0.94	0.90	0.89
	2050	0.98	0.95	0.85	0.95	0.94	**0.60**	0.99	**0.32**	**0.47**	**0.59**	**0.43**	0.89	0.91	0.79

BE, Belgium; DE, Denmark; FR, France; GE, Germany; IR, Ireland; IT, Italy; NE, the Netherlands; NO, Norway; PO, Portugal; SP, Spain; SWE, Sweden; SWI, Switzerland, UK, the United Kingdom; EU, studied area (15°W, 20°E, 35°N, 60°N). The average SI for countries marked with an asterisk must be treated with caution because the calibration (SDMs‐NAEU) was performed only including very limited parts of their territories. Bold text indicates changes of over 10% between current and future climate conditions.

**Figure 3:**
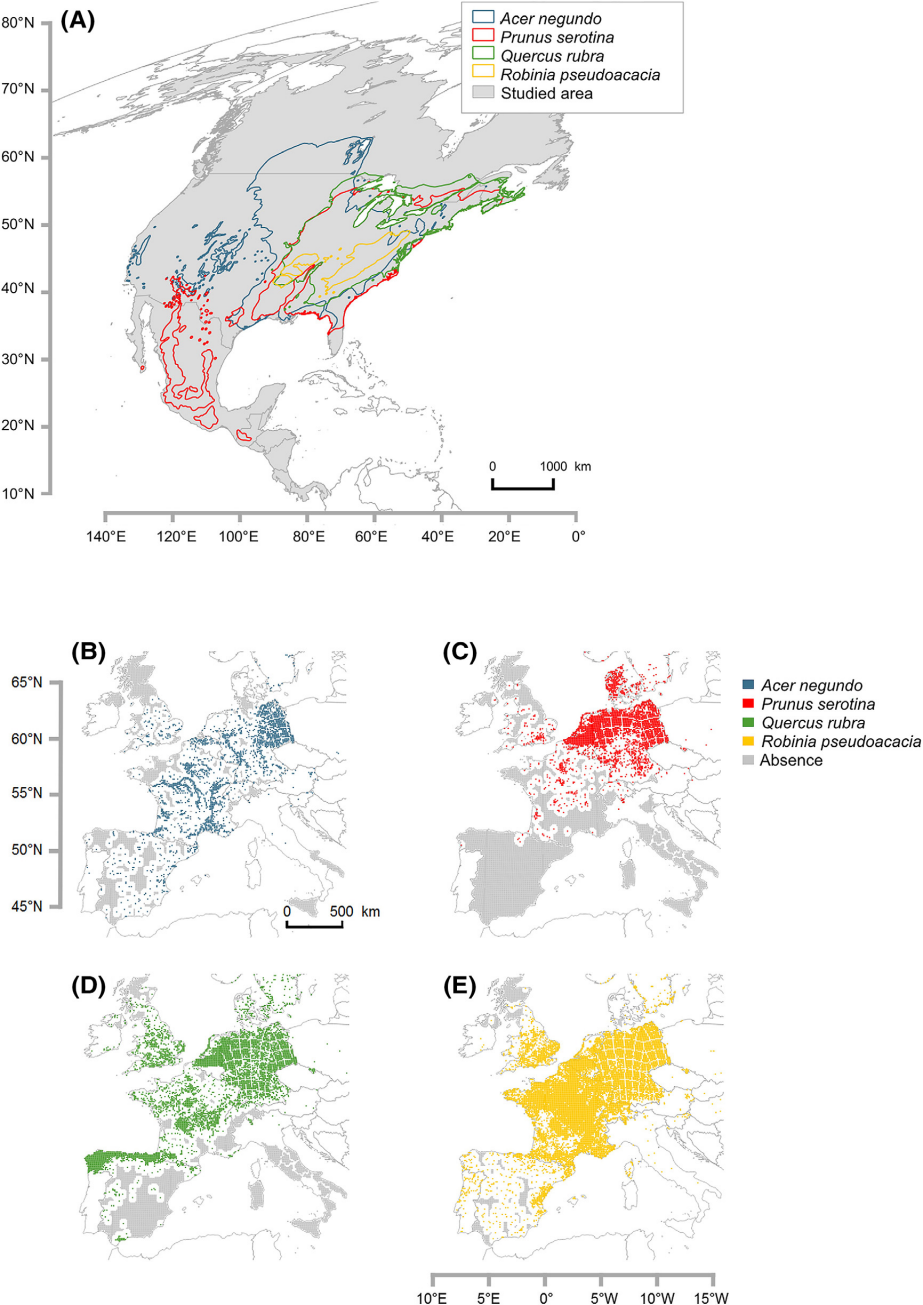
Maps of the native ranges of the four species in North America (A) and presence data in Western Europe for each species: *Acer negundo* in blue (B), *Prunus serotina* in red (C), *Quercus rubra* in green (D), and *Robinia pseudoacacia* in yellow (E). In the native range (A), gray indicates the background used in the study corresponding to the North American geographical boundaries excluding the Tundra biome (Olson et al. 2001). In the invasive range, gray corresponds to pseudo‐absence data created with a buffer zone (white) around presence data with well‐established spatial coverage (France, Spain, the United Kingdom, Flanders, the Netherlands, and Germany) or to true absence (in Italy). Countries that are not included in the analysis are presented in white. Data sources are presented in Table S2.

Under the current climate, SI was high for *R. pseudoacacia* over the entire study area, being <0.5 only for a few parts of southern Spain, Portugal, and Italy, in Scotland, and in the eastern part of the Alps in Austria and Slovenia. This result is generally consistent with observations (Fig. 3), but contrasts markedly with the observed occurrences in Spain, where only a few populations are currently found in central Spain. The projection under future climatic conditions indicated no change in suitability over the temperate regions, but a large reduction in suitable habitat over the whole Mediterranean region: Only the North Atlantic coast of Spain and the interior mountains of Italy (Apennines) would still be suitable for this species in 2050.

The distribution of a suitable habitat over Western Europe under the current climate is quite similar for *A. negundo*, but with lower suitability in central Spain and Portugal, in Ireland, and the United Kingdom, and in the Alps in general. However, this suitable habitat is largely unfilled (Figs. 1, 3) because natural populations mostly occur along riverbanks under current conditions. By 2050, moderate changes were observed in habitat suitability: The projection mainly suggests an increase in the low suitability in the already unsuitable habitats of Spain, Portugal, and Italy. In contrast, the Alps and the United Kingdom would present more suitable habitats.

Under current climatic conditions, habitat suitability for *Q. rubra* and *P. serotina* was high in the temperate regions of Western Europe; the Mediterranean region presented low suitability for *Q. rubra* (SI < 0.5 in most areas) and was associated with a low probability of occurrence (SI < 0.3) for *P. serotina*. Overall, these patterns corresponded to the observed occurrences of the species in Western Europe (Fig. 3), although they suggested large geographical unfilling in France and Scotland, particularly for *P. serotina*. Projections in 2050 suggested a northward shift in habitat suitability for both species. For *P. serotina*, almost all of Spain, Portugal, and Italy were predicted to become unsuitable and large losses in habitat suitability were also predicted in France. For *Q. rubra*, the pattern of habitat suitability across Europe reflected an accentuation of unsuitability in southern and central Spain and Italy, as well as a moderate loss of habitat suitability in southwest France.

## Discussion

Any assessment of the risk of species invasion must be preceded by an assessment of the niche conservatism between the native and invasive ranges (Petitpierre et al. 2012). Here, we applied a number of complementary techniques to assess the degree of niche shift (Warren et al. 2008; Aguirre‐Gutiérrez et al. 2015), overlapping (Guisan et al. 2014), and transferability (Fernández and Hamilton 2015), for four invasive trees from North America to Europe. We estimated the climatic risk of invasibility of those species in Western Europe in the present and also in the future.

### Changes in climatic niches

Native and invasive ranges show low‐to‐moderate overlap for all species, reflecting differences in climate niches between North America and Western Europe. For *A. negundo* and *P. serotina*, niches are neither interchangeable nor similar, whereas for *Q. rubra* and *R. pseudoacacia*, significant niche similarity test results suggest that their Western European niches have more climatic characteristics in common with their North American ones than expected randomly, suggesting that other drivers than climatic ones are probably limiting their distributions in Western Europe. Low transferability of SDMs calibrated with the invasive or native ranges were previously reported for *A. negundo* and *P. serotina* (Petitpierre et al. 2012), suggesting again that realized rather than fundamental niches are shaping the invasibility of the species in Western Europe.

Biotic interactions could explain niche differences apart from climate (EICA hypothesis; Blossey and Notzold 1995). Indeed, in its invasive range, *A. negundo* is more common in riparian ecosystems, whereas it grows in mixed broad‐leaved forests in Canada (Lamarque et al. 2012). This niche shift can be explained by the fact that *A. negundo* can outcompete both the pioneer and the late successional native tree species only in light‐ and nitrogen‐rich environments (Porté et al. 2011), matching the conditions found in these ecosystems that are subject to substantial perturbation and management by humans (Richardson et al. 2007). The unfilling observed for *A. negundo* could also result from local adaptation during the lag phase (Lee 2002; Prentis et al. 2008). Indeed, *A. negundo* invasive populations show higher phenotypic diversity and genetic differentiation than native ones (Lamarque et al. 2015) and local adaptation followed by natural environmental colonization was previously shown for *A. negundo* (Erfmeier et al. 2011). Similarly, some populations of *P. serotina* located in France and England were found to be genetically different from native ones (Pairon et al. 2010). Genetic differentiation and niche shift in invasive species can be related to a founder effect (Sax et al. 2007), although in forestry multiple introductions are often suspected (Donaldson et al. 2014). Due to the likelihood of multiple introductions, it was difficult to conclude that there were any genetic differences in *Q. rubra* between native and invasive populations (Magni et al. 2005). Still, the invasive populations of *Q. rubra* seemed to be characterized by a greater plasticity with regard to growth and phenology (Petit et al. 2004). *Robinia pseudoacacia* is known to have undergone increased selection for use in forestry in Central Europe (Rédei et al. 2008, 2011), with low between‐population variability being observed in this region (Liesebach et al. 2004), but the genetic differentiation between populations in the native and introduced ranges remains poorly understood. Therefore, larger provenance trials and new molecular markers are required to include phenotypic variations due to genetic differentiation in SDMs (Benito Garzón et al. 2011; Valladares et al. 2014).

### Present and future invasion risks by North American trees in Western Europe

Overall, invasive species currently occupy only half of their potential distribution ranges (Gasso et al. 2012), which can be estimated by niche unfilling, a good estimate of the likely expansion in the near future in the invasive ranges. For the four species studied here, niche unfilling is associated with some regions of France, the United Kingdom, and Spain, whereas in Central Europe, the species have already covered all their potential ranges for invasion. Because these species are already considered highly invasive in Central Europe (Enescu and Dănescu 2013; Woziwoda et al. 2014), efforts are needed to minimize their expansion in Western Europe, where the species have not yet fully expressed their invasiveness and could potentially spread more.

Locally, *P. serotina* and *R. pseudoacacia* can expand through root sucker production. Seeds of *R. pseudoacacia* and *A. negundo* are dispersed by wind and water, whereas those of *Q. rubra* and *P. serotina* are mostly dispersed by animals; these differences could contribute to the differences in the realized niches of the species. In particular, *R. pseudoacacia,* having both sexual and asexual spreading abilities, presents very low niche unfilling, so invasion risks are high all over the studied range. The projections made using SDM calibrated for the native and invasive ranges identify *unfilling* in Southern Europe, where the species is less common and could eventually expand its range under the current conditions. In its native range, *R. pseudoacacia* is characterized by a very restricted distribution range because of competition for light with other trees, in combination with pathogen damage (Burns and Honkala 1990). After colonizing a new site, this pioneer species is replaced by more competitive trees after 15–30 years (Cierjacks et al. 2013). However, in its introduced range, the lack of natural regulators and its regrowth capacity after wood harvesting give it a unique advantage in disrupted environments, which can lead to the exclusion of native species (Enescu and Dănescu 2013). The high climate suitability in the present and future conditions for *R. pseudoacacia* suggests a risk of expansion of this species in Europe.

All species present very low suitability in southern Spain and Italy, but the *Robinia* and *Acer* species are actually already present in southern Spain. This mismatch between the model and the data could reflect the fact that these populations are found in artificial plantations installed regardless of climate suitability (Donaldson et al. 2014). Indeed, the acclimation and natural spread of these temperate North American trees are known to be difficult in these regions because of their lower adaptation to the arid conditions (Gasso et al. 2012; González‐Muñoz et al. 2015). Our models project a likely shift of the suitable climatic range northwards for all of the species in the future climate (of 2050). Hence, in the near future, all species will present lower risks in these southern regions (of Spain and Italy) and the management needed for the four invasive species could be reconsidered in drier parts of Europe. For *P. serotina*, this potential reduction in invasion risk could also occur in the west and south of France. At the same time, in northern areas such as in the United Kingdom, the potential increased risks presented by *A. negundo* and *P. serotina* under climate change should be considered.

## Perspectives and conclusions

We focused our study on Western Europe, one of the areas invaded by four North American tree species, but we are also aware that these four species are also invading other parts of the world (e.g., Australia, New Zealand, South Africa, South America, Pacific Islands, and even non‐native areas of North America). Therefore, our analysis can be applied to other invasive ranges of the species, including for comparative purposes.

The differences in climate‐related invasion risks between the present and the future as shown by our models need to be taken into account by stakeholders in order to establish adaptive management plans.

## Conflict of Interest

None declared.

## Supporting information


**Figure S1A.** Coefficient of variation between an averaging model and 11 Global Climatic Models for their variable “Temperature Seasonality” by using RCP4.0 and 2050 period (2040–2060).
**Figure S1B.** Coefficient of variation between an averaging model and 11 Global Climatic Models for their variable “Mean Temperature of the Warmest Month” by using RCP4.0 and 2050 period (2040–2060).
**Figure S1C.** Coefficient of variation between an averaging model and 11 Global Climatic Models for their variable “Mean Temperature of the Coldest Month” by using RCP4.0 and 2050 period (2040–2060).
**Figure S1D.** Coefficient of variation between an averaging model and 11 Global Climatic Models for their variable “Precipitation Seasonality” by using RCP4.0 and 2050 period (2040–2060).
**Figure S1E.** Coefficient of variation between an averaging model and 11 Global Climatic Models for their variable “Precipitation of the Driest Quarter” by using RCP4.0 and 2050 period (2040–2060).
**Figure SIF.** Coefficient of variation between an averaging model and 11 Global Climatic Models for their variable “Precipitation of the Warmest Quarter” by using RCP4.0 and 2050 period (2040–2060).
**Figure S1G.** Coefficient of variation between an averaging model and 11 Global Climatic Models for their variable “Precipitation of the Coldest Quarter” by using RCP4.0 and 2050 period (2040–2060).
**Figure S2.** Evaluating model transferability from Europe to North America: habitat suitability under current climate conditions (1950–2000) for the studied species over North America using model SDM‐EU calibrated using Western Europe presence/absence data.
**Figure S3.** Evaluating model transferability from North America to Europe: habitat suitability under current climate conditions (1950–2000) for the studied species in Western Europe using model SMD‐NA calibrated using presence/absence data from North America.
**Table S1.** Spearman's correlation between climatic variables for current period.
**Table S2A.** Databases used for building presence/ absence data set in Western Europe.
**Table S2B.** Algorithms used for the development of the SDMs.
**Table S3A.** Performance of SDMs calibrated on presence/ absence data from the native range (North America).
**Table S3B.** Performance of SDMs calibrated on *P*/*A* data in West Europe. For details, see table S3.
**Table S3C.** Performance of SDMs calibrated using data from West Europe and North America. For details, see table S3A.
**Table S4.** Relative importance of the climate variables on the SDMs‐NAEU.
