# Supplementary material for: American trees shift their niches when invading Western Europe: evaluating invasion risks in a changing climate
Source: Ecol Evol. 2016 Sep 22;6(20):7263–75. doi: 10.1002/ece3.2376 (PMC5513278; doi:10.1002/ece3.2376)
Supplement: Supplementary file 1 — Figure S1A. Coefficient of variation between an averaging model and 11 Global Climatic Models for their variable “Temperature Seasonality” by using RCP4.0 and 2050 period (2040–2060). Figure S1B. Coefficient of variation between an averaging model and 11 Global Climatic Models for their variable “Mean Temperature of the Warmest Month” by using RCP4.0 and 2050 period (2040–2060). Figure S1C. Coefficient of variation between an averaging model and 11 Global Climatic Models for their variable “Mean Temperature of the Coldest Month” by using RCP4.0 and 2050 period (2040–2060). Figure S1D. Coefficient of variation between an averaging model and 11 Global Climatic Models for their variable “Precipitation Seasonality” by using RCP4.0 and 2050 period (2040–2060). Figure S1E. Coefficient of variation between an averaging model and 11 Global Climatic Models for their variable “Precipitation of the Driest Quarter” by using RCP4.0 and 2050 period (2040–2060). Figure SIF. Coefficient of variation between an averaging model and 11 Global Climatic Models for their variable “Precipitation of the Warmest Quarter” by using RCP4.0 and 2050 period (2040–2060). Figure S1G. Coefficient of variation between an averaging model and 11 Global Climatic Models for their variable “Precipitation of the Coldest Quarter” by using RCP4.0 and 2050 period (2040–2060). Figure S2. Evaluating model transferability from Europe to North America: habitat suitability under current climate conditions (1950–2000) for the studied species over North America using model SDM‐EU calibrated using Western Europe presence/absence data. Figure S3. Evaluating model transferability from North America to Europe: habitat suitability under current climate conditions (1950–2000) for the studied species in Western Europe using model SMD‐NA calibrated using presence/absence data from North America. Table S1. Spearman's correlation between climatic variables for current period. Table S2A. Databases used for building presence/ [file ECE3-6-7263-s001.docx]

**Supporting Information**

**American trees shift their niches when invading Western Europe: Evaluating invasion risks in a changing climate**

Etienne Camenen, Annabel J. Porté* and Marta Benito Garzón

*Correspondence: A J. Porté E-mail: annabel.porte@u-bordeaux.fr


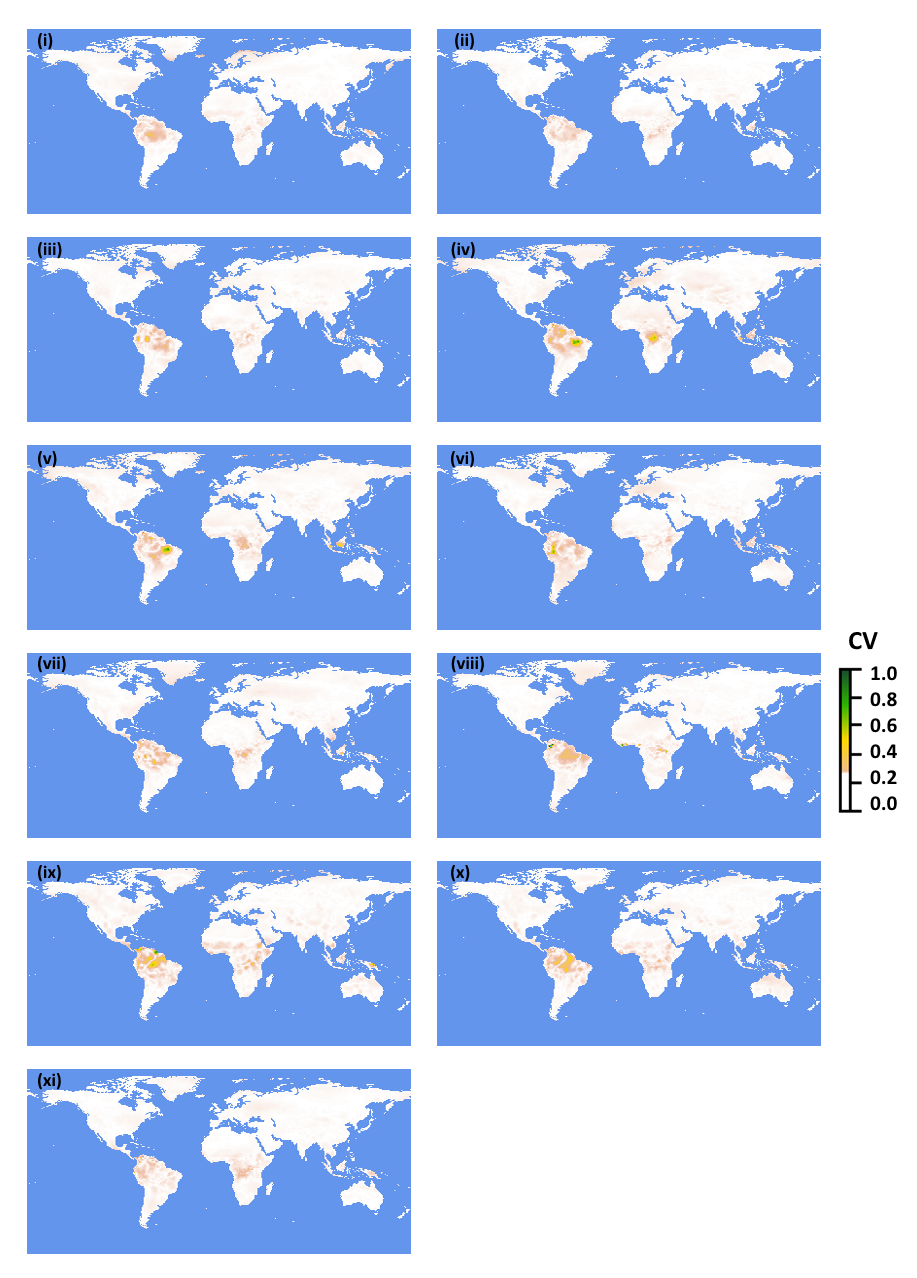
Fig S1A. Coefficient of variation between an averaging model and 11 Global Climatic Models for their variable « Temperature Seasonality » by using RCP4.0 and 2050 period (2040-2060). *(i)* BCC-CSM1-1; *(ii)* CCSM4; *(iii)* GISS-E2-R; (iv) HadGEM2-AO; (v) HadGEM2-ES; (vi) IPSL-CM5A-LR; (vii) MIROC5; (viii) MRI-CGCM3; (ix) MIROC-ESM-CHEM; (x) MIROC-ESM et (xi) NorESM1-M.


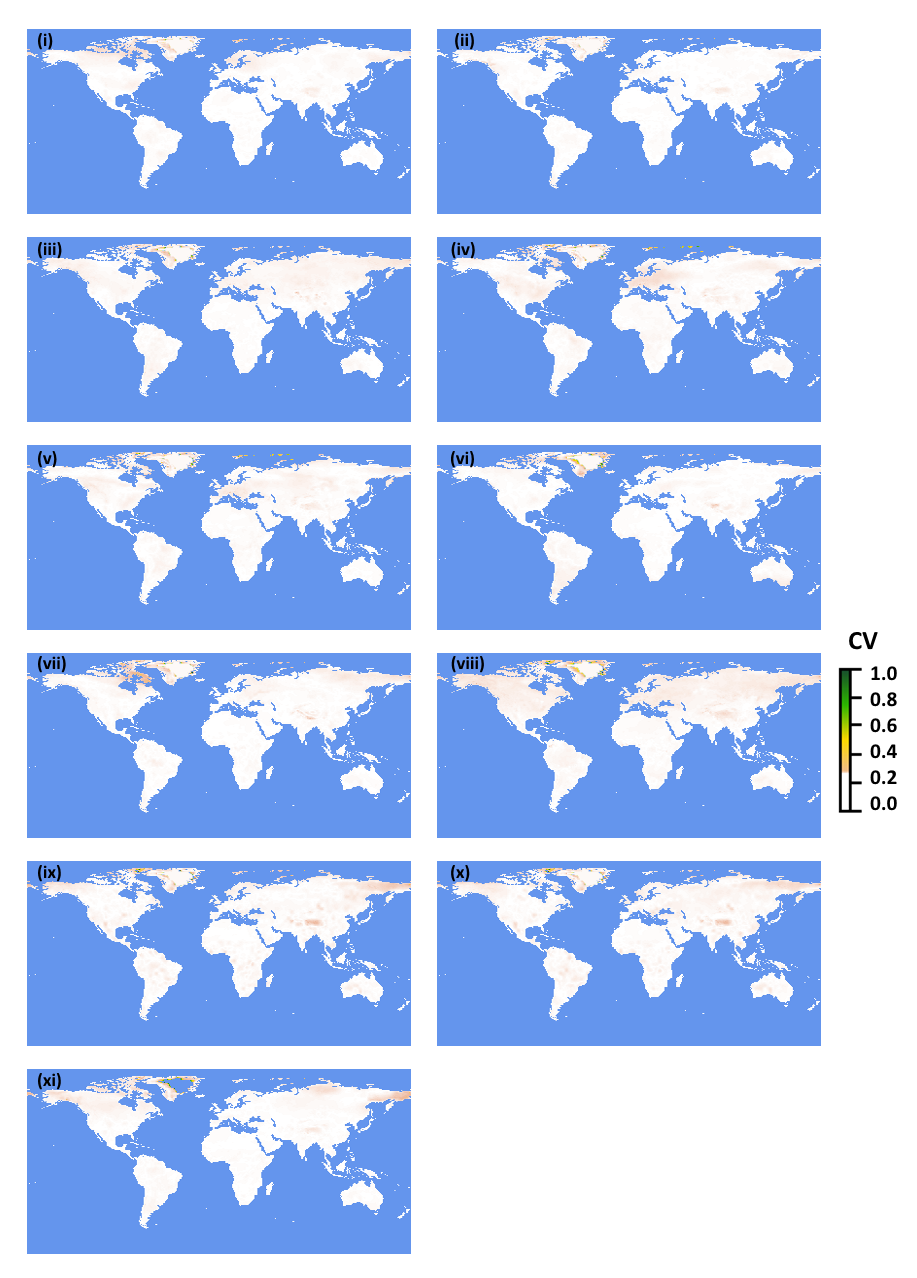
Fig. S1B. Coefficient of variation between an averaging model and 11 Global Climatic Models for their variable « Mean Temperature of the Warmest Month » by using RCP4.0 and 2050 period (2040-2060). *(i)* BCC-CSM1-1; *(ii)* CCSM4; *(iii)* GISS-E2-R; (iv) HadGEM2-AO; (v) HadGEM2-ES; (vi) IPSL-CM5A-LR; (vii) MIROC5; (viii) MRI-CGCM3; (ix) MIROC-ESM-CHEM; (x) MIROC-ESM et (xi) NorESM1-M.


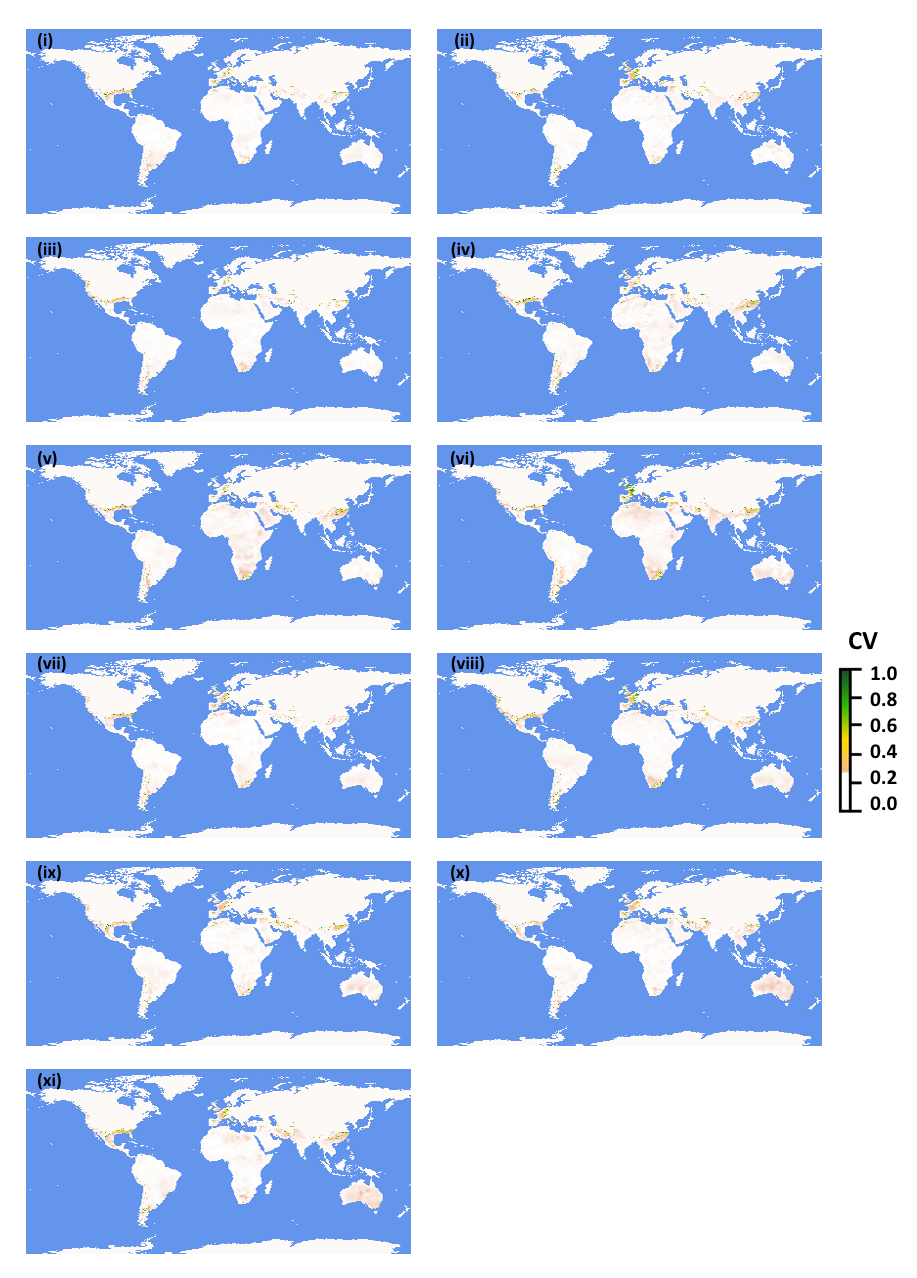
Fig. S1C. Coefficient of variation between an averaging model and 11 Global Climatic Models for their variable « Mean Temperature of the Coldest Month » by using RCP4.0 and 2050 period (2040-2060). *(i)* BCC-CSM1-1; *(ii)* CCSM4; *(iii)* GISS-E2-R; (iv) HadGEM2-AO; (v) HadGEM2-ES; (vi) IPSL-CM5A-LR; (vii) MIROC5; (viii) MRI-CGCM3; (ix) MIROC-ESM-CHEM; (x) MIROC-ESM et (xi) NorESM1-M..


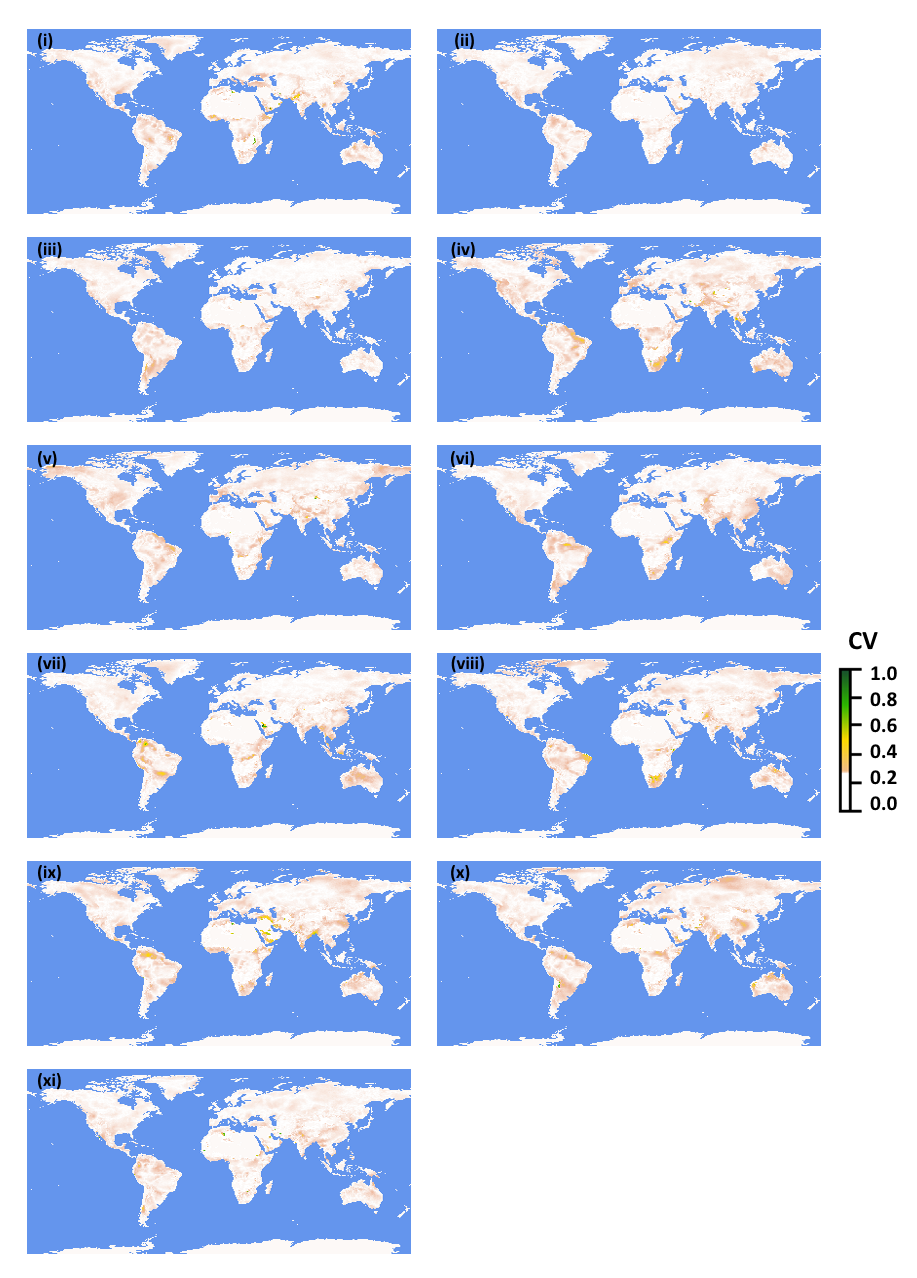
Fig. S1D. Coefficient of variation between an averaging model and 11 Global Climatic Models for their variable « Precipitation Seasonality » by using RCP4.0 and 2050 period (2040-2060). *(i)* BCC-CSM1-1; *(ii)* CCSM4; *(iii)* GISS-E2-R; (iv) HadGEM2-AO; (v) HadGEM2-ES; (vi) IPSL-CM5A-LR; (vii) MIROC5; (viii) MRI-CGCM3; (ix) MIROC-ESM-CHEM; (x) MIROC-ESM et (xi) NorESM1-M.


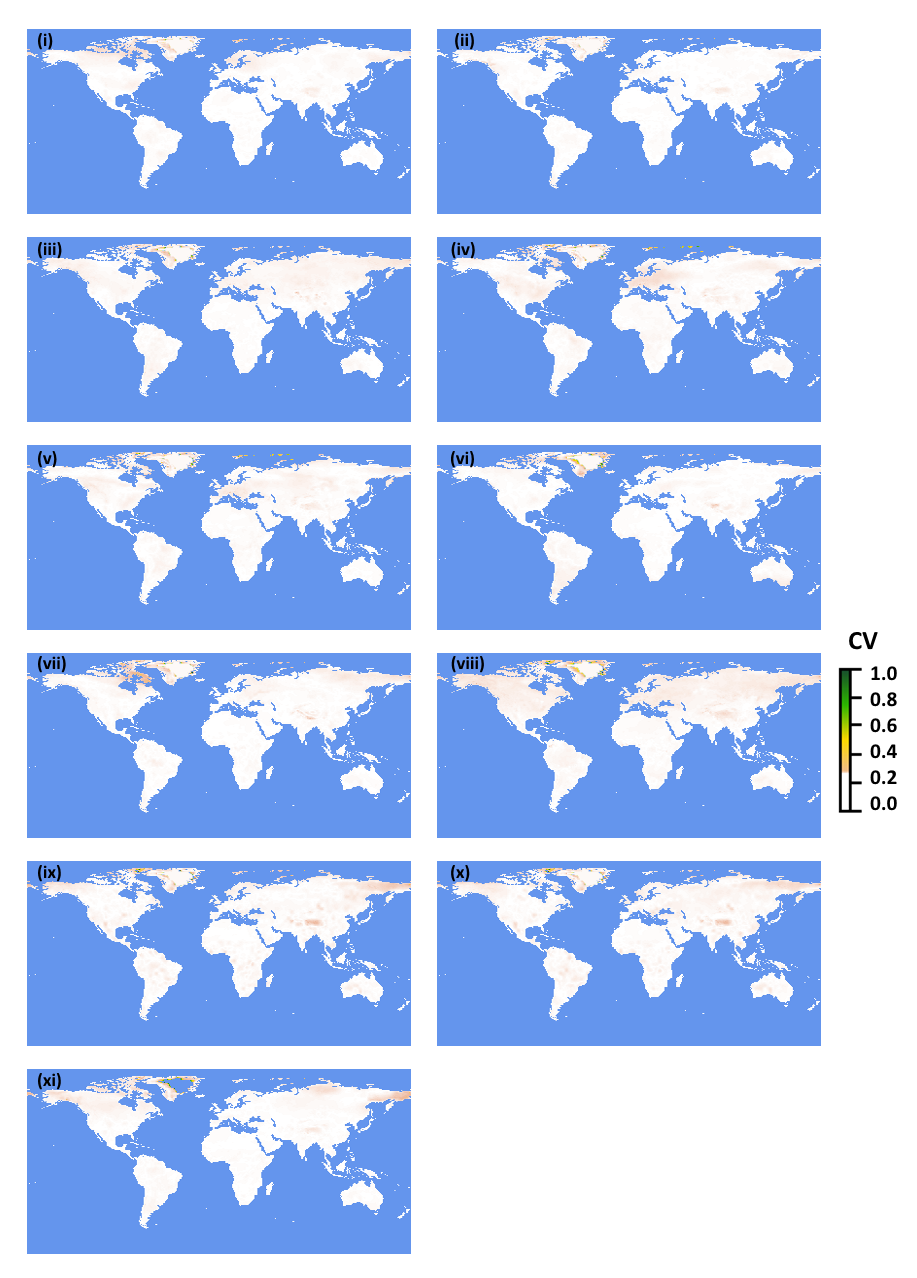
Fig. S1E. Coefficient of variation between an averaging model and 11 Global Climatic Models for their variable « Precipitation of the Driest Quarter» by using RCP4.0 and 2050 period (2040-2060). *(i)* BCC-CSM1-1; *(ii)* CCSM4; *(iii)* GISS-E2-R; (iv) HadGEM2-AO; (v) HadGEM2-ES; (vi) IPSL-CM5A-LR; (vii) MIROC5; (viii) MRI-CGCM3; (ix) MIROC-ESM-CHEM; (x) MIROC-ESM et (xi) NorESM1-M..


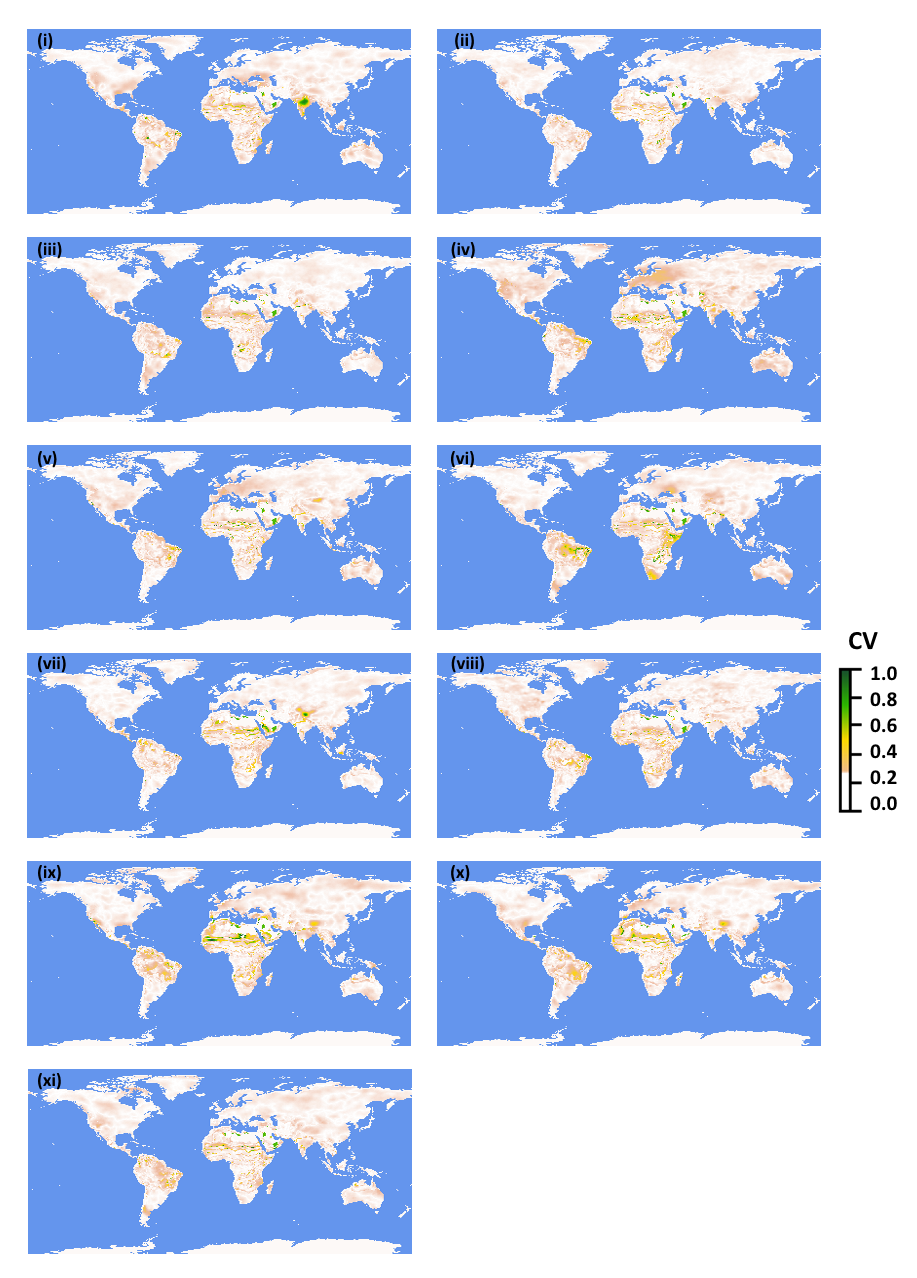
Fig. SIF. Coefficient of variation between an averaging model and 11 Global Climatic Models for their variable « Precipitation of the Warmest Quarter » by using RCP4.0 and 2050 period (2040-2060). *(i)* BCC-CSM1-1; *(ii)* CCSM4; *(iii)* GISS-E2-R; (iv) HadGEM2-AO; (v) HadGEM2-ES; (vi) IPSL-CM5A-LR; (vii) MIROC5; (viii) MRI-CGCM3; (ix) MIROC-ESM-CHEM; (x) MIROC-ESM et (xi) NorESM1-M..


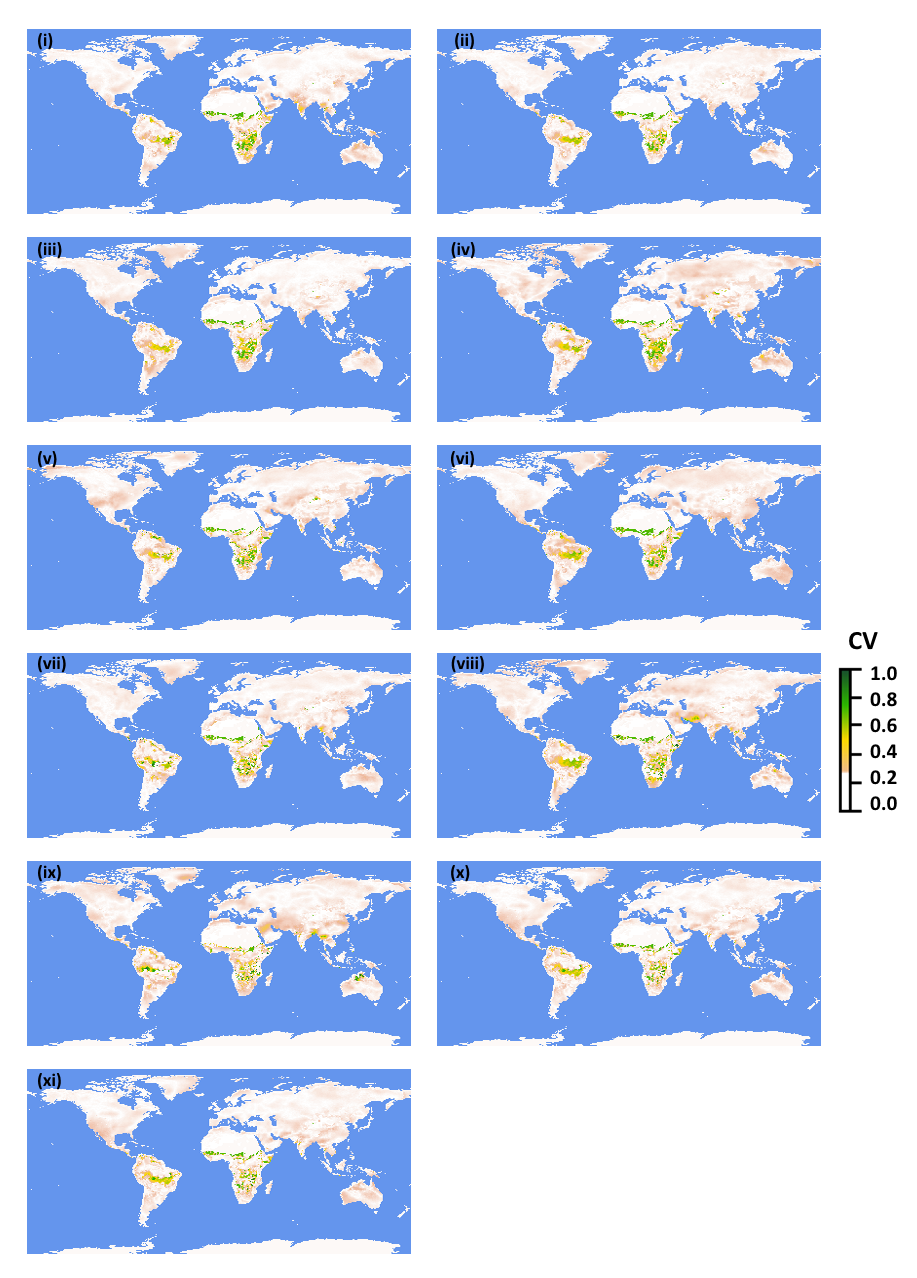
Fig. S1G. Coefficient of variation between an averaging model and 11 Global Climatic Models for their variable « Precipitation of the Coldest Quarter » by using RCP4.0 and 2050 period (2040-2060). *(i)* BCC-CSM1-1; *(ii)* CCSM4; *(iii)* GISS-E2-R; (iv) HadGEM2-AO; (v) HadGEM2-ES; (vi) IPSL-CM5A-LR; (vii) MIROC5; (viii) MRI-CGCM3; (ix) MIROC-ESM-CHEM; (x) MIROC-ESM et (xi) NorESM1-M.

Fig. S2 Evaluating model transferability from Europe to North America: habitat suitability under current climate conditions (1950-2000) for the studied species over North America using model SDM-EU calibrated using Western Europe presence/absence data
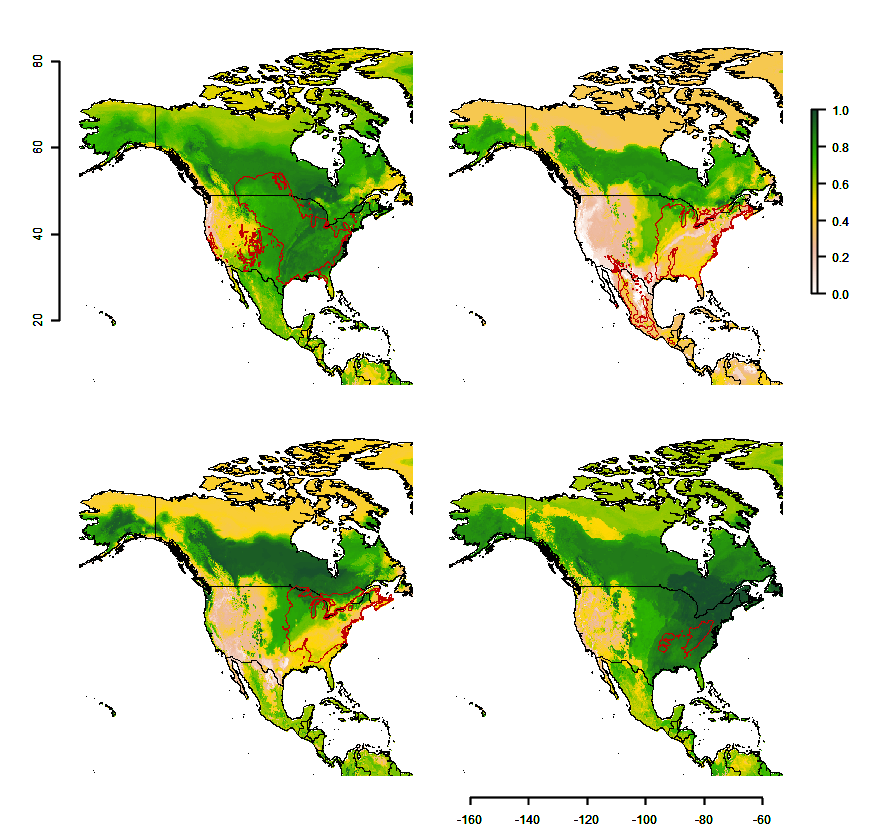
. (a) *Acer negundo*; (b) *Prunus serotina*; (c) *Quercus rubra*; (d) *Robinia pseudoacacia*. Dark green colors indicate high suitability, yellow colors indicate moderate suitability and light red colors indicate low suitability. Red shapes indicate native range as described by Little (1971).

**(a) (b)**

**(c) (d)**

Fig. S3 Evaluating model transferability from North America to Europe: habitat suitability under current climate conditions (1950-2000) for the studied species in Western Europe using model SMD-NA calibrated using presence/absence data from North America
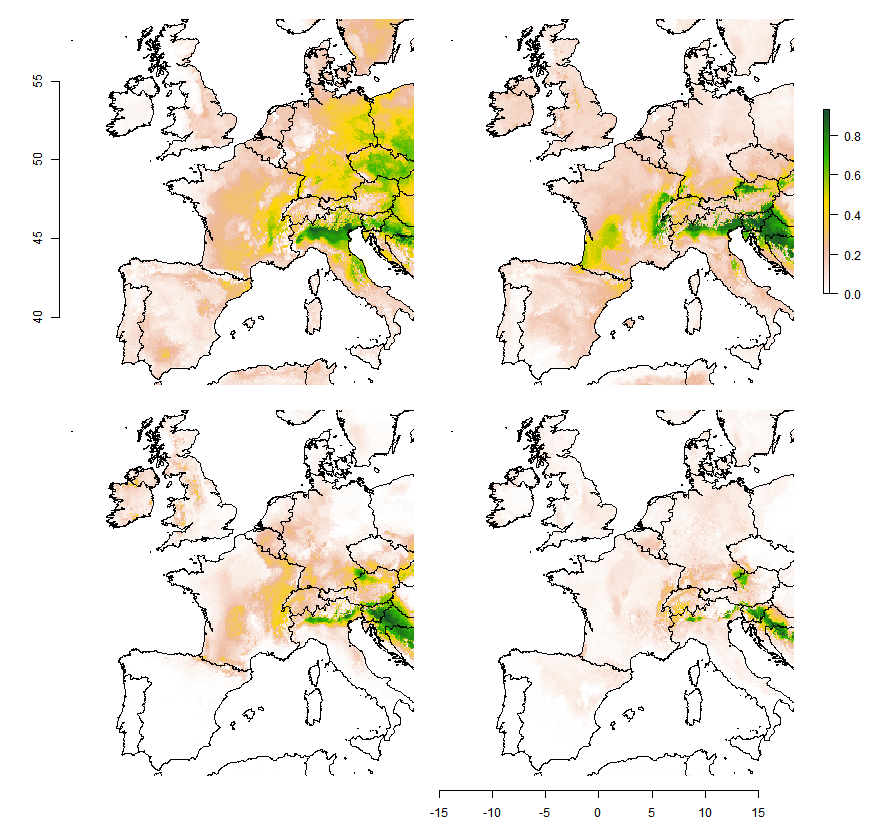
. (a) *Acer negundo;* (b) *Prunus serotina;* (c) *Quercus rubra;* (d) *Robinia pseudoacacia.* Dark green colors indicate high suitability, yellow colors indicate moderate suitability and light red colors indicate low suitability.

**(a) (b)**

**(c) (d)**

Table S1. Spearman’s correlation between climatic variables for current period. PS = precipitation seasonality; PDQ = mean precipitation of the driest quarter; PWQ = mean precipitation of the warmest quarter; PCQ = mean precipitation of the coldest quarter; TS = temperature seasonality; MTWM = mean temperature of the warmest month; MTCM = mean temperature of the coldest month.

|  | PS | PDQ | PWQ | PCQ | TS | MTWM | MTCM |
| --- | --- | --- | --- | --- | --- | --- | --- |
| North America |  |  |  |  |  |  |  |
| PS | - | - | - | - | - | - | - |
|  |  |  |  |  |  |  |  |
|  |  |  |  |  |  |  |  |
| PDQ | -0.759 | - | - | - | - | - | - |
| PWQ | -0.253 | 0.667 | - | - | - | - | - |
| PCQ | -0.641 | 0.862 | 0.521 | - | - | - | - |
| TS | -0.067 | -0.151 | -0.174 | -0.381 | - | - | - |
| MTWM | 0.163 | -0.125 | 0.087 | -0.083 | -0.607 | - | - |
| MTCM | 0.057 | 0.120 | 0.225 | 0.313 | -0.948 | 0.793 | - |
| Western Europe |  |  |  |  |  |  |  |
| PS | - | - | - | - | - | - | - |
| PDQ | -0.768 | - | - | - | - | - | - |
| PWQ | -0.141 | 0.601 | - | - | - | - | - |
| PCQ | -0.630 | 0.846 | 0.426 | - | - | - | - |
| TS | -0.160 | -0.061 | -0.261 | -0.225 | - | - | - |
| MTWM | 0.406 | -0.363 | -0.119 | -0.152 | -0.579 | - | - |
| MTCM | 0.244 | -0.060 | 0.185 | 0.155 | -0.938 | 0.787 | - |

Table S2A. Databases used for building presence/ absence data set in Western Europe.

| **Name** | **Information** | **Website** |
| --- | --- | --- |
| Anthos | Spanish plant information system, Spain | [www.anthos.es](http://www.anthos.es) |
|  |  |  |
| FNCB | National federation of botanic conservatories, France | http://siflore.fcbn.fr/ |
|  |  |  |
| GBIF | Global Biodiversity Information Facilities; international open data infrastructure, funded by governments. | [www.gbif.org](http://www.gbif.org)  [www.gbif.es](http://www.gbif.es) |
|  |  |  |
| IFN France | National Forest Inventory for France | [www.inventaire-forestier.ign.fr](http://www.inventaire-forestier.ign.fr) |
|  |  |  |
| IFN Spain | National Forest Inventory for Spain | [www.magrama.gob.es/es/biodiversidad/servicios/banco-datos-naturaleza/informacion-disponible/ifn3.aspx](http://www.magrama.gob.es/es/biodiversidad/servicios/banco-datos-naturaleza/informacion-disponible/ifn3.aspx) |
|  |  |  |
| Info Flora | National data and information center on Swiss flora, Switzerland | https://www.infoflora.ch/fr/flore/recherche-par-esp%C3%A8ce.html |

Table S2B. Algorithms used for the development of the SDMs. GLM, General Linear Model; GAM, General Additive Mode, CART (Classification And Regression Trees); MRT, Multivariate Regression Trees; SVM, Support Vector Machine; GBM, Boosted Regression Trees; RF, Random Forests. The correspondent R library and main parameters are indicated as well as reference for more details.

| Methods | Library | Parameters | | References |
| --- | --- | --- | --- | --- |
| GLM | {stats} | Quadratic formula, backward & forward selection (stepAIC {MASS}) | |  |
| GAM | {mgcv} | family="binomial",select=TRUE,method="REML" | |  |
| CART | {rpart} |  |  |  |
| MRT | {mvpart} | xv="1se", xvmult=50 | |  |
| SVM | {e1071} |  |  |  |
| GBM | {gbm} | n.trees=2000,interaction.depth=2,cv.folds=10 | |  |
| RF | {randomForest} |  |  |  |

Table S3A. Performance of SDMs calibrated on presence/ absence data from the native range (North America). Bold characters indicate the highest performance coefficients. Pearson correlation (“PC(SD)”) between observed P/A values and predicted values (on 1/3 of the initial dataset which was not used for model calibration) was used to assess the generalization power of the model with the standard deviation averaged for 10 runs of models . True Skill Statistics (“TSS”) evaluate the match between the habitat suitability prediction and the occurrence of the species; it ranges from -1 to 1 from the worst to the best match, respectively. The Suitability Index threshold allows the conversion from SI continuous maps to binary maps. The values showed correspond to the SI threshold chosen to optimize TSS’ values are averaged on the results of 10 SDMs’ runs (TOT). For algorithm details, see Table S1.

|  | **PC** | **Goodness-of-fit** | | **EU Transferability** | |
| --- | --- | --- | --- | --- | --- |
|  |  | **TSS** | **SI threshold** | **Se** | **SI** |
| ***A. negundo*** |  |  |  |  |  |
| **GLM** | 0.85(0) | 0.85(0) | 0.91(0) | 0.05(0) | 0.50(0) |
| **GAM** | 0.88(0) | 0.85(0) | 0.92(0) | 0.05(0) | 0.50(0) |
| **CART** | 0.87(0) | 0.82(0.02) | 0.90(0.03) | 0.22(0.16) | 0.70(0.17) |
| **MRT** | 0.87(0) | 0.82(0.01) | 0.89(0.02) | 0.10(0.13) | 0.86(0.14) |
| **SVM** | 0.91(0) | 0.90(0) | 0.90(0.01) | 0.60(0.02) | 0.5(0) |
| **GBM** | 0.80(0) | 0.75(0) | 0.78(0) | 0.16(0.01) | 0.50(0) |
| **RF** | **0.97(0)** | **0.97(0)** | **0.97(0)** | **0.97(0)** | **0.97(0)** |
| **TOT** | 0.87(0) | 0.84(0.01) | 0.90(0.01) | 0.26(0.05) | 0.67(0.05) |
| ***P. serotina*** |  |  |  |  |  |
| **GLM** | 0.89(0) | 0.86(0) | **0.96(0)** | **0.69(0.02)** | 0.50(0) |
| **GAM** | 0.92(0) | 0.87(0) | 0.95(0) | 0.65(0.03) | 0.50(0) |
| **CART** | 0.88(0.01) | 0.81(0) | **0.96(0)** | 0.16(0.11) | **0.68(0.10)** |
| **MRT** | 0.92(0) | 0.87(0) | 0.95(0) | 0.65(0.03) | 0.50(0) |
| **SVM** | 0.93(0) | 0.90(0) | **0.96(0)** | 0.05(0.01) | 0.50(0) |
| **GBM** | 0.87(0) | 0.86(0) | 0.82(0.01) | 0.03(0) | 0.50(0) |
| **RF** | **0.98(0)** | **0.98(0)** | 0.90(0.01) | 0.02(0) | 0.50(0) |
| **TOT** | 0.91(0) | 0.88(0) | 0.92(0) | 0.31(0.03) | 0.54(0.02) |
| ***Q. rubra*** |  |  |  |  |  |
| **GLM** | 0.96(0) | 0.94(0) | **0.97(0)** | 0.15(0.01) | 0.50(0) |
| **GAM** | 0.97(0) | 0.95(0) | 0.95(0) | **0.49(0.08)** | 0.50(0) |
| **CART** | 0.93(0) | 0.82(0.01) | 0.96(0) | 0.04(0.01) | **0.96(0)** |
| **MRT** | 0.97(0) | 0.95(0) | 0.95(0) | **0.49(0.08)** | 0.50(0) |
| **SVM** | 0.96(0) | 0.94(0) | 0.93(0.01) | 0.11(0.02) | 0.5(0) |
| **GBM** | 0.91(0) | 0.88(0) | 0.85(0.01) | 0.07(0) | 0.50(0) |
| **RF** | **0.99(0)** | **0.98(0)** | 0.94(0.01) | 0.01(0) | 0.50(0) |
| **TOT** | 0.95(0) | 0.91(0) | 0.93(0) | 0.15(0.02) | 0.59(0) |
| ***R. pseudoacacia*** | |  |  |  |  |
| **GLM** | 0.97(0) | 0.85(0.04) | 0.99(0) | 0.09(0.06) | 0.50(0) |
| **GAM** | 0.97(0) | 0.86(0.01) | 0.99(0) | 0.06(0.02) | 0.50(0) |
| **CART** | 0.95(0.01) | 0.41(0.01) | 0.96(0) | 0.13(0.06) | **0.92(0.09)** |
| **MRT** | 0.97(0) | 0.86(0.01) | 0.99(0) | 0.06(0.02) | 0.50(0) |
| **SVM** | 0.95(0) | 0.81(0.02) | **1.00(0)** | 0.09(0.01) | 0.5(0) |
| **GBM** | 0.94(0) | 0.48(0.01) | 0.86(0.01) | **0.19(0.01)** | 0.50(0) |
| **RF** | **0.98(0)** | **0.96(0.01)** | **1.00(0)** | 0(0) | 0.51(0.01) |
| **TOT** | 0.96(0) | 0.71(0.02) | 0.96(0) | 0.09(0.03) | 0.59(0.02) |

Table S3B. Performance of SDMs calibrated on P/A data in West Europe. For details, see table S3

|  | **PC** | **Goodness-of-fit** | | **NA Transferability** | |
| --- | --- | --- | --- | --- | --- |
|  |  | **TSS** | **SI** | **TSS** | **SI** |
| ***A. negundo*** |  |  |  |  |  |
| **GLM** | 0.78(0.01) | 0.88(0) | 0.50(0) | 0.21(0.01) | 0.94(0.05) |
| **GAM** | 0.82(0.01) | 0.89(0) | 0.50(0) | 0.28(0.09) | 0.92(0.10) |
| **CART** | 0.79(0.02) | 0.86(0.02) | **0.64(0.10)** | 0.40(0.14) | 0.94(0.04) |
| **MRT** | 0.78(0.01) | 0.86(0.04) | 0.61(0.11) | 0.35(0.05) | 0.91(0.10) |
| **SVM** | 0.82(0.01) | 0.90(0) | 0.50(0) | 0.56(0.11) | 0.98(0.03) |
| **GBM** | 0.76(0.01) | 0.86(0.01) | 0.50(0) | 0.39(0.01) | 0.76(0.07) |
| **RF** | **0.90(0.01)** | **0.97(0)** | **0.50(0)** | 0.26(0.02) | 0.67(0.04) |
| **TOT** | 0.81(0.01) | 0.89(0.01) | 0.54(0.03) | 0.35(0.06) | 0.87(0.06) |
| ***P. serotina*** |  |  |  |  |  |
| **GLM** | 0.90(0.01) | 0.94(0) | 0.50(0) | -0.18(0) | 1.00(0) |
| **GAM** | 0.91(0) | 0.94(0) | 0.50(0) | -0.13(0.10) | 0.84(0.24) |
| **CART** | 0.87(0.01) | 0.91(0.01) | **0.87(0.12)** | -0.17(0.02) | 0.99(0.02) |
| **MRT** | 0.86(0.01) | 0.92(0.01) | 0.84(0.13) | -0.18(0) | 1.00(0) |
| **SVM** | 0.91(0.01) | 0.95(0) | 0.50(0) | -0.15(0.03) | 0.55(0.16) |
| **GBM** | 0.86(0.01) | 0.93(0.01) | 0.50(0) | -0.18(0) | 1.00(0) |
| **RF** | **0.95(0)** | **0.99(0)** | 0.50(0) | -0.16(0.02) | 0.80(0.14) |
| **TOT** | 0.89(0.01) | 0.94(0) | 0.60(0.04) | -0.16(0.02) | 0.88(0.08) |
| ***Q. rubra*** |  |  |  |  |  |
| **GLM** | 0.89(0) | 0.96(0) | 0.50(0) | 0.01(0.01) | 0.50(0) |
| **GAM** | 0.92(0.01) | 0.97(0) | 0.50(0) | 0.17(0.01) | 0.70(0.23) |
| **CART** | 0.87(0.01) | 0.95(0.01) | 0.77(0.11) | 0.26(0.06) | 0.78(0.12) |
| **MRT** | 0.87(0.01) | 0.94(0.01) | **0.83(0.10)** | 0.10(0.18) | 0.84(0.10) |
| **SVM** | 0.82(0.01) | 0.90(0) | 0.50(0) | 0.56(0.11) | 0.98(0.03) |
| **GBM** | 0.86(0.01) | 0.95(0) | 0.50(0) | -0.04(0) | 0.62(0.08) |
| **RF** | **0.96(0)** | **0.99(0)** | 0.50(0) | 0.10(0.08) | 0.50(0) |
| **TOT** | 0.88(0.01) | 0.95(0) | 0.59(0.03) | 0.17(0.06) | 0.70(0.08) |
| ***R. pseudoacacia*** | |  |  |  |  |
| **GLM** | 0.92(0.01) | 0.94(0.01) | 0.50(0) | 0.02(0.01) | 0.50(0.01) |
| **GAM** | **0.96(0.01)** | **0.97(0)** | 0.50(0) | 0.02(0.01) | 0.93(0.15) |
| **CART** | 0.91(0.02) | 0.96(0.01) | 0.78(0.15) | 0.05(0.03) | 0.97(0.01) |
| **MRT** | 0.90(0.02) | 0.96(0.02) | **0.86(0.08)** | 0.03(0) | 0.95(0.01) |
| **SVM** | 0.93(0.01) | 0.96(0) | 0.50(0) | 0.17(0.04) | 0.70(0.07) |
| **GBM** | 0.88(0.02) | 0.91(0) | 0.50(0) | 0.16(0.02) | 0.88(0.02) |
| **RF** | **0.96(0.01)** | **0.97(0)** | 0.50(0) | 0.09(0.01) | 0.93(0.04) |
| **TOT** | 0.92(0.01) | 0.95(0.01) | 0.59(0.03) | 0.08(0.02) | 0.84(0.04) |

Table S3C. Performance of SDMs calibrated using data from West Europe and North America. For details, see table S3A

|  | **PC** | **Goodness-of-fit** | | **EU Transferability** | | **NA Transferability** | | | |
| --- | --- | --- | --- | --- | --- | --- | --- | --- | --- |
|  |  | **TSS** | **SI** | **Se** | **SI** | **TSS** | | **SI** | |
| ***A. negundo*** | | | | | | | | | |
| **GLM** | 0.83(0) | 0.67(0) | 0.83(0.09) | 0.66(0) | 0.83(0.09) | | 0.99(0) | | 0.50(0) |
| **GAM** | 0.88(0.01) | 0.80(0.01) | **0.87(0.02)** | 0.80(0.01) | 0.87(0.02) | | 0.98(0) | | 0.51(0.01) |
| **CART** | 0.87(0.01) | 0.80(0.02) | **0.87(0.05)** | 0.79(0.02) | 0.87(0.05) | | 0.95(0.01) | | 0.74(0.20) |
| **MRT** | 0.87(0.01) | 0.80(0.02) | 0.86(0.04) | 0.79(0.02) | 0.86(0.04) | | 0.95(0.02) | | 0.80(0.17) |
| **SVM** | 0.91(0) | 0.89(0.01) | 0.82(0.02) | 0.89(0.01) | 0.83(0.02) | | 0.99(0) | | 0.51(0.01) |
| **GBM** | 0.78(0.01) | 0.75(0.04) | 0.78(0.04) | 0.68(0.01) | 0.74(0.05) | | 0.96(0) | | 0.50(0) |
| **RF** | **0.97(0.01)** | **0.94(0)** | 0.83(0.12) | 0.94(0.03) | 0.79(0.01) | | 1.00(0.14) | | 0.52(0) |
| **TOT** | 0.87(0.01) | 0.81(0.01) | 0.84(0.05) | 0.79(0.01) | 0.83(0.04) | | 0.97(0.02) | | 0.58(0.06) |
| ***P. serotina*** | | | | | | | | | |
| **GLM** | 0.91(0.01) | 0.86(0) | 0.95(0.01) | 0.86(0) | 0.96(0.01) | | 1.00(0) | | 0.59(0.03) |
| **GAM** | 0.93(0) | 0.88(0.01) | 0.90(0.02) | 0.87(0.01) | 0.90(0.02) | | 1.00(0) | | 0.58(0.07) |
| **CART** | 0.90(0.01) | 0.80(0.01) | 0.95(0.04) | 0.80(0.01) | 0.95(0.04) | | 0.99(0.01) | | 0.82(0.08) |
| **MRT** | 0.89(0.01) | 0.79(0.02) | **0.96(0.03)** | 0.78(0.02) | 0.96(0.03) | | 0.98(0.01) | | 0.76(0.13) |
| **SVM** | 0.94(0.01) | 0.89(0) | 0.94(0.05) | 0.89(0) | 0.94(0.05) | | 1.00(0) | | 0.52(0.02) |
| **GBM** | 0.86(0.01) | 0.82(0.01) | 0.84(0.02) | 0.82(0.02) | 0.85(0.02) | | 1.00(0) | | 0.51(0.01) |
| **RF** | **0.96(0.10)** | **0.94(0)** | 0.84(0) | 0.94(0.05) | 0.86(0.01) | | 1.00(0) | | 0.56(0.01) |
| **TOT** | 0.91(0.02) | 0.85(0.01) | 0.91(0.02) | 0.85(0.02) | 0.92(0.03) | | 1.00(0) | | 0.62(0.05) |
| ***Q. rubra*** |  |  |  |  |  | |  | |  |
| **GLM** | 0.93(0) | 0.91(0) | **0.95(0.01)** | 0.90(0) | 0.96(0.01) | | 0.98(0) | | 0.50(0) |
| **GAM** | 0.97(0) | 0.93(0) | 0.91(0.01) | 0.93(0) | 0.92(0.01) | | 0.99(0) | | 0.50(0.01) |
| **CART** | 0.92(0.01) | 0.84(0.03) | 0.91(0.02) | 0.82(0.03) | 0.91(0.02) | | 0.94(0.01) | | 0.91(0.12) |
| **MRT** | 0.91(0.01) | 0.82(0.02) | 0.92(0.01) | 0.81(0.02) | 0.91(0.02) | | 0.94(0.01) | | 0.94(0.06) |
| **SVM** | 0.96(0) | 0.94(0.01) | 0.89(0.01) | 0.94(0.01) | 0.90(0.02) | | 1.00(0) | | 0.51(0.01) |
| **GBM** | 0.84(0.01) | 0.87(0) | 0.82(0.01) | 0.88(0) | 0.82(0.01) | | 0.98(0) | | 0.50(0) |
| **RF** | **0.98(0.05)** | **0.96(0)** | 0.89(0.07) | 0.97(0) | 0.88(0.01) | | 1.00(0.09) | | 0.51(0.04) |
| **TOT** | 0.93(0.01) | 0.90(0.01) | 0.90(0.02) | 0.89(0.01) | 0.90(0.01) | | 0.98(0.02) | | 0.62(0.03) |
| ***R. pseudoacacia*** | | | | | | | | | |
| **GLM** | 0.92(0) | 0.96(0) | 0.99(0) | 0.63(0.05) | 0.99(0) | | 0.98(0) | | 0.50(0) |
| **GAM** | 0.95(0) | 0.93(0.01) | 0.99(0) | 0.73(0.02) | 0.98(0.01) | | 0.99(0) | | 0.50(0) |
| **CART** | 0.92(0.01) | 0.57(0.02) | 0.96(0) | 0.35(0.02) | 0.95(0.02) | | 0.95(0.02) | | 0.80(0.17) |
| **MRT** | 0.92(0.01) | 0.58(0.06) | 0.96(0.01) | 0.36(0.04) | 0.95(0.02) | | 0.95(0.03) | | 0.77(0.21) |
| **SVM** | 0.95(0) | 0.93(0.01) | **1.00(0)** | 0.86(0.02) | 1.00(0) | | 1.00(0) | | 0.50(0) |
| **GBM** | 0.90(0.01) | 0.83(0) | 0.86(0.01) | 0.64(0.02) | 0.87(0) | | 0.95(0) | | 0.50(0) |
| **RF** | **0.99(0.01)** | **0.97(0.01)** | 0.99(0.06) | 0.98(0) | 0.99(0) | | 1.00(0) | | 0.51(0.10) |
| **TOT** | 0.94(0.01) | 0.82(0.02) | 0.96(0.01) | 0.65(0.02) | 0.96(0.01) | | 0.97(0.01) | | 0.58(0.07) |

Table S4. Relative importance of the climate variables on the SDMs-NAEU. MTCM: Mean Temperature of the Coldest Month; MTWM: Mean Temperature of the Warmest Month; PCQ: Precipitation of the Coldest Quarter; PDQ: Precipitation of the Driest Quarter; PS: Precipitation Seasonality; PWQ: Precipitation of the Warmest Quarter; TS: Temperature Seasonality. Gray and red light to dark colors correspond to low to high values.

| **Species** | **TS** | **MTWM** | **MTCM** | **PS** | **PDQ** | **PWQ** | **PCQ** |
| --- | --- | --- | --- | --- | --- | --- | --- |
| ***A. negundo*** | 32.6 | 54.9 | 52.5 | 43.3 | 27.5 | 36 | 28.6 |
| ***P. serrotina*** | 26.5 | 45.3 | 51 | 38.8 | 24.3 | 51.1 | 19.4 |
| ***Q. rubra*** | 37.6 | 59 | 59.6 | 49.3 | 36.1 | 54.4 | 18.3 |
| ***R. pseudoaccia*** | 55.9 | 46.2 | 64.4 | 48.8 | 32.2 | 38.8 | 22.7 |
| **Mean** | 38.2 | 51.4 | 56.9 | 45.1 | 30.0 | 45.1 | 22.3 |
| **SD** | 12.7 | 6.7 | 6.3 | 5.0 | 5.2 | 9.0 | 4.6 |

# SUPPLEMENT METHDOLOGY

# References

Broennimann O.. Fitzpatrick M.C.. Pearman P.B.. Petitpierre B.. Pellissier L.. Yoccoz N.G.. Thuiller W.. Fortin M.-J.. Randin C.. Zimmermann N.E.. & others (2012) Measuring ecological niche overlap from occurrence and spatial environmental data. *Global Ecology and Biogeography*. **21**. 481–497.

Broennimann O.. Treier U.A.. Müller-Schärer H.. Thuiller W.. Peterson A.T.. & Guisan A. (2007) Evidence of climatic niche shift during biological invasion. *Ecology Letters*. **10**. 701–709.

Guisan A.. Petitpierre B.. Broennimann O.. Daehler C.. & Kueffer C. (2014) Unifying niche shift studies: insights from biological invasions. *Trends in ecology & evolution*. **29**. 260–269.

Petitpierre B.. Kueffer C.. Broennimann O.. Randin C.. Daehler C.. & Guisan A. (2012) Climatic niche shifts are rare among terrestrial plant invaders. *Science*. **335**. 1344–1348.

Schoener T.W. (1968) The Anolis lizards of Bimini: resource partitioning in a complex fauna. *Ecology*. **49**. 704–726.

Silverman B.W. (1986) *Density estimation for statistics and data analysis.* CRC press.

Van Vuuren D.P.. Edmonds J.. Kainuma M.. Riahi K.. Thomson A.. Hibbard K.. Hurtt G.C.. Kram T.. Krey V.. Lamarque J.-F.. & others (2011) The representative concentration pathways: an overview. *Climatic Change*. **109**. 5–31.

Warren D.L.. Glor R.E.. & Turelli M. (2008) Environmental niche equivalency versus conservatism: quantitative approaches to niche evolution. *Evolution*. **62**. 2868–2883.

Worton B.J. (1989) Kernel methods for estimating the utilization distribution in home-range studies. *Ecology*. **70**. 164–168.
